# Regulation of amyloid-β levels by matrix metalloproteinase-2/9 (MMP2/9) in the media of lung cancer cells

**DOI:** 10.1038/s41598-021-88574-0

**Published:** 2021-05-06

**Authors:** Sadaf Dorandish, Asana Williams, Sarah Atali, Sophia Sendo, Deanna Price, Colton Thompson, Jeffrey Guthrie, Deborah Heyl, Hedeel Guy Evans

**Affiliations:** Chemistry Department, Eastern Michigan University, Ypsilanti, MI 48197 USA

**Keywords:** Cancer, Cell biology

## Abstract

In this study, we set out to identify regulators of intact amyloid-β40/42 (Aβ) levels in A549 (p53 wild-type) and H1299 (p53-null) lung cancer cell media. Higher Aβ levels were detected in the media of A549 than H1299 cells without or with treatment with 4-methylumbelliferone (4-MU) and/or the anti-CD44 antibody (5F12). Using inhibitors, we found that PI3K, AKT, and NFκB are likely involved in regulating Aβ levels in the media. However, increased Aβ levels that more closely resembled those found upon 4-MU co-treatment resulted from MMP2/9 inhibition, suggesting that MMP2/9 maybe the main contributors to regulation of Aβ levels in the media. Differences in Aβ levels might be accounted for, in part, by p53 since blocking p53 function in A549 cells resulted in decreased Aβ levels, increased MMP2/9 levels, increased PI3K/AKT activities and the phospho/total NFκB ratio. Using siRNA targeted against MMP2 or MMP9, we found increased Aβ levels in the media, however, MMP2 knockdown led to Aβ levels closely mimicking those detected by co-treatment with 4-MU. Cell viability or apoptosis upon treatment with either MMP2 or MMP9 siRNA along with Aβ immunodepletion, showed that MMP2 is the predominant regulator of the cytotoxic effects induced by Aβ in lung cancer cells.

## Introduction

Despite extensive research, lung cancer has a poor prognosis for patients with metastatic disease and remains the leading cause of cancer-related deaths with an approximate 15% 5-year survival rate in the United States and worldwide^1,2^. Excluding small cell carcinoma, non-small cell lung carcinoma (NSCLC) includes adenocarcinoma, squamous cell carcinoma, and large cell carcinoma^3^.

Amyloid-beta (Aβ) is a peptide produced by numerous types of cells and is widely accepted to play a role in the pathogenesis of Alzheimer’s disease (AD)^4–8^. The ~ 4 kDa Aβ peptide is derived from the sequential processing of the higher molecular weight amyloid precursor protein (APP)^4,9^. APP is an integral membrane protein that produces Aβ upon sequential cleavage by two membrane-bound endoproteases, β- and γ‐secretase^5,7,8,10^. Cleavage by γ-secretase removes the remaining C-terminal fragment of the transmembrane domain of APP, liberating Aβ from the cellular membrane into the extracellular space^11–13^. The somewhat imprecise cleavage by γ-secretase results in C-terminal heterogeneity and different Aβ species with Aβ40 being the most abundant (~ 80–90%), followed by Aβ42 (~ 5–10%) known to be more hydrophobic and fibrillogenic, and the main species deposited in the AD brain^4,9^. APP is also known to be cleaved by α-secretase, an activity mainly attributed to the ADAM (a disintegrin and metalloprotease domain) family of proteases, such as ADAM 9, 10, 17, and 19^14–16^. The metalloproteinase ADAM10 is a key protease involved in the non-amyloidogenic pathway^14–16^. ADAM10 prevents Aβ generation by cleaving APP within the Aβ domain generating a carboxy-terminal fragment that resides in the membrane and a secreted soluble ectodomain of APP, sAPPα, with neuroprotective and neurotrophic properties^14^.

Hyaluronic acid (HA) is a nonsulfated glycosaminoglycan that along with its family members composed of HA receptors (CD44, cluster of differentiation antigen 44; RHAMM, hyaluronan-mediated motility receptor), HA synthases (HAS1, HAS2, HAS3), and hyaluronidases, promotes tumor growth and progression^17–27^. HA along with its receptor, CD44, upregulates tumor cell proliferation and survival^17–27^ by activating intracellular signaling^23,28,29^. HA is known to regulate several cellular functions and its expression has been reported to be elevated in a variety of tumors^22,30^. Each of the three HAS synthesizes different molecular weights of HA and silencing HAS genes in tumors is known to block cell proliferation and metastasis^17,30^. Both high levels of CD44 and HA are emerging as important metastatic markers in a wide range of human carcinomas^17,18,20,22–24,26,26^.

The antitumor activity of the dietary supplement, orally bioavailable, coumarin derivative, and relatively non-toxic drug, 4-methylumbelliferone (4-MU), is thought to be primarily due to inhibition of HA synthesis^31–34^. In mammalian cells, HA is synthesized from UDP-N-acetyl-D-glucosamine and UDP-glucuronic acid (UGA), a substrate for UDP-glucuronosyltransferases^19,30,31^. Upon treatment with 4-MU, glucuronic acid is transferred onto 4-MU by UDP-glucuronosyltransferases resulting in depletion of the UGA intracellular pool, blocking HA synthesis^19,22,23^.

Phosphoinositide 3 Kinase (PI3K) catalyzes formation of the lipid second messenger phosphatidylinositol-3,4,5-triphosphate (PIP3) leading to recruitment and activation of downstream targets that include the serine/threonine protein kinase, AKT, known to be activated by lipid products of PI3K^35,36^. The PI3K/AKT signaling pathway, known to be dysregulated in a number of human cancers, results in phosphorylation of numerous protein targets and regulates a wide range of cellular processes critical for tumorigenesis including proliferation, survival, and growth^35,37^. Activation of PI3K/AKT signaling was found to attenuate Aβ-induced apoptosis via downregulation of GSK-3β, known to increase hyperphosphorylation of the tau protein and neurofibrillary tangles formation^38,39^. Aβ was shown to inhibit the PI3K pathway in neuronal cells inducing neurotoxicity, while activation of the PI3K pathway using a direct PI3K activator, resulted in neuroprotective effects in Aβ-induced neuronal cell death^40^.

In response to a range of stress signals, the p53 tumor suppressor protein regulates the expression of an array of various genes that subsequently mediate the p53 response, including those involved in induction of apoptosis and senescence, cell-cycle arrest, and blocking cell proliferation^41,42^. High expression of CD44 in many tumor malignancies is related to the onset of tumor progression, and via counteracting p53 tumor-suppressor function, can nourish growth and survival of cancer cells in various stages of progression^43,44^. Conversely, the tumor suppressor, p53, acts to repress CD44 expression leading to cell-cycle arrest and cell death through apoptosis, and antiproliferative activities against cancer development^43,44^.

The binding of HA to CD44 is also known to induce the expression and activity of the large family of matrix metalloproteinases (MMPs), highly homologous, Zn^2+^-dependent endopeptidases, involved in degradation of extracellular matrix components and tumor progression^45^. Normally, MMPs are synthesized as latent pro-enzymes that can become fully active upon proteolytic processing^46,47^.

In this study, we used two human NSCLC cell lines^48^, A549 (p53-positive) and H1299 (p53-null)^49^ to gain further mechanistic insights into regulation of Aβ40/42 levels in the media of lung cancer cells. Based on the literature, we hypothesized that increased extracellular full-length intact Aβ40/42 concentrations, via a mechanism that involves downregulating signaling of PI3K/AKT, NFκB, and MMP2/ 9, lead to cytotoxic effects in lung cancer cells in a p53-dependent manner.

## Results

### More Aβ is detected in the media of A549 and H1299 cells upon treatment with either 4-MU and/or 5F12

**Figure 1:**
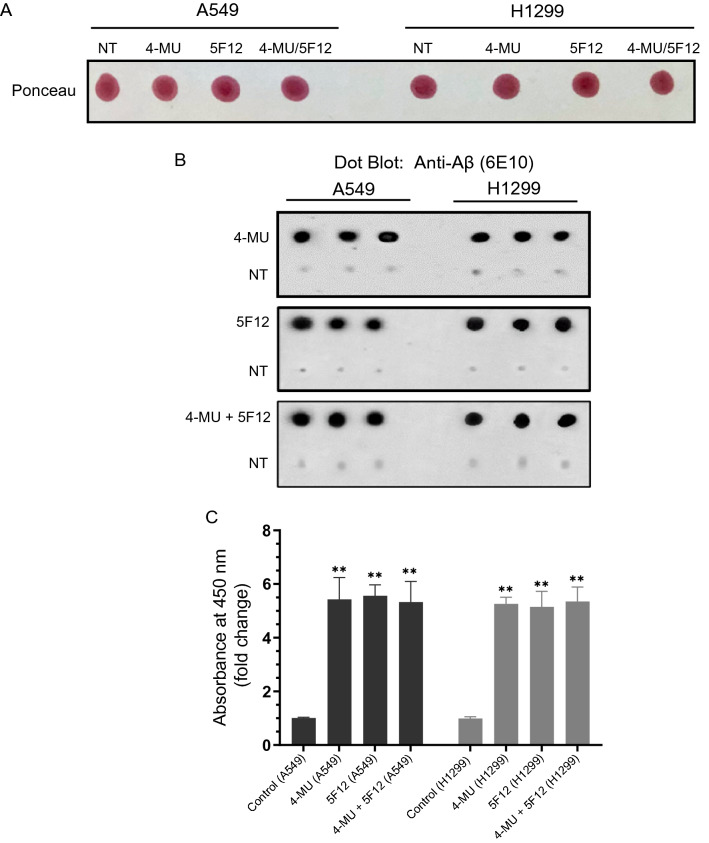
Treatment of cells with either 4-MU or 5F12 resulted in increased Aβ in the media of A549 and H1299 cells. Cells (0.2 × 10^5^) were grown in 10% FBS-supplemented media for 24 h. The following day, the cell monolayers were incubated in serum-free media for 24 h, then treated as indicated for 72 h with 600 µM 4-MU and/or the CD44 antibody (5F12, 5 μg/mL). The media was collected then the same amount of protein (3 µL of 600 µg/mL total protein) of each sample not-treated (NT) or treated as indicated, was spotted onto a nitrocellulose membrane. The blots were stained with Ponceau (**A**). (**B**) Blots were incubated with anti-Aβ (6E10) antibodies, and the signal on the membrane was detected using super signal west pico luminol (chemiluminescence) reagent. The membranes were imaged with a Bio-Rad molecular imager, and quantitated with Image J. The dots from five independent assays, each carried out in triplicate, were quantitated, averaged, normalized, and expressed as fold change relative to untreated control cells after subtraction of the values for the blank consisting of dot blots probed with mouse IgG isotype control with no relevant specificity to a target antigen (mIgG, 5 μg/mL) or anti-Aβ (6E10) antibodies incubated with blots dotted with media not incubated with cells (**C**) using the GraphPad 8.4.3 software. The graphs summarize the results expressed as means ± SD (n = 5). Asterisks (*) indicate a statistically significant difference from the corresponding cell line control, **p < 0.0l of each cell line, Mann–Whitney test.

Previously, we found that the Insulin-Like Growth Factor Binding Protein-3, IGFBP-3, blocks HA-CD44 interactions by binding to HA, leading to decreased survival of A549 (p53 wild-type) lung cancer cells^50^. We subsequently showed that blocking HA-CD44 signaling by IGFBP-3 resulted in increased levels of acetylcholinesterase in A549 cell media but not in the media of H1299 (p53-null) lung cancer cells, effects that correlated with a greater reduction in A549 cell viability^51^. We also found that the relative abundance of Aβ oligomer versus total Aβ increased upon immunodepletion of the cytoprotective peptide, humanin, from the conditioned media of A549 and H1299 cells, leading to increased apoptosis and diminished cell viability^52^. More recently, we showed that ATP, thought to decrease misfolding of Aβ, strengthened interactions between humanin and Aβ but weakened those between Aβ and acetylcholinesterase^53^. More humanin was bound to Aβ upon ATP addition to media from either A549 or H1299 lung cancer cells, while reduced acetylcholinesterase levels were found in a complex with Aβ by ATP addition to A549 cell media^53^. To better understand the mechanisms regulating the levels of Aβ in the media, A549 and H1299 cells were grown in FBS-supplemented media for 24 h, then incubated in serum-free media overnight. The cells were then treated as indicated with 4-MU and/or the CD44 antibody (Fig. 1) and the media collected. Blots were incubated (Fig. 1B) with anti-Aβ specific antibodies (6E10), known to react with monomers, oligomers and fibrils of Aβ^54,55^ and recognize the N-terminal hydrophilic Aβ sequence, amino acids 1–16. This epitope, is known to be exposed in Aβ aggregates^54^, and using a high-resolution mapping approach, 6E10 was found to more specifically map to amino acid residues 4–10^56^.

Consistent with our previous report^57^, the levels of Aβ were comparable in the media of both cell lines (Fig. 1) measured by using anti-Aβ 6E10 antibodies. These Aβ levels in the conditioned media of both cell lines were higher upon treatment of cells with either 4-MU and/or 5F12 (Fig. 1). With each treatment, the levels of Aβ were increased ~ 5.5-fold relative to untreated cell control (Fig. 1C). Since 4-MU is thought to exert its effects primarily due to inhibition of HA synthesis^31–34^, and as the anti-CD44 antibody (5F12) is known to be antagonistic towards HA-CD44 molecular interactions^58,59^, the observed comparable levels of Aβ in the media of either cell line upon cell treatment with 4-MU and/or 5F12 suggest that the increased Aβ levels might result from disruption of HA signaling. The 6E10 antibodies are known to recognize the N-terminal hydrophilic sequence, amino acids 1–16 of Aβ^54,55,60^, and this epitope has been reported to be exposed in Aβ aggregates^54^. Therefore, the signal obtained from using this antibody is most likely a representation of any Aβ fragment containing this region which might include Aβ1-x, Aβ1-42 or Aβ1-40.

### The levels of intact Aβ40 and Aβ42 are higher in the media of A549 cells as compared to that of H1299 cells

Substantial evidence indicates that Aβ solubility, quantity in different pools, and self-assembly of Aβ40/42 peptides into amyloid fibrils, are factors associated with what is now known to be greater than 20 devastating human disorders, including AD and other serious neurodegenerative diseases^6–8,10,61–63^. Oligomers form more readily from Aβ42, than the more abundant Aβ40, which has lower aggregation kinetics and toxicity than Aβ42^7^. The C-terminus of Aβ42 is known to be critical for oligomer formation, and amyloid deposition is thought to be driven almost entirely by Aβ42 rather than Aβ40^64^. The N-terminal region of the Aβ sequence is hydrophilic, while nearly all hydrophobic amino acids make up the C-terminal part, proposed to account for its propensity to misfold and aggregate^65^.

Plasma levels of Aβ40 and Aβ42 in pancreatic and breast cancer tumors have been detected and also reported to be higher in all cancer patients, including those with colorectal, esophageal, hepatic, and lung cancers, than in normal controls^66,67^. Moreover, Aβ was shown to accumulate naturally in glioma tumors and in culture, and glioma cells were found to produce and release the Aβ40 peptide into the media that acted to block human U87 glioblastoma subcutaneous xenografts in nude mice^67,68^. In addition, overexpression of Aβ40 in transgenic mice led to reductions in glioma growth and invasion^67,69^.

**Figure 2:**
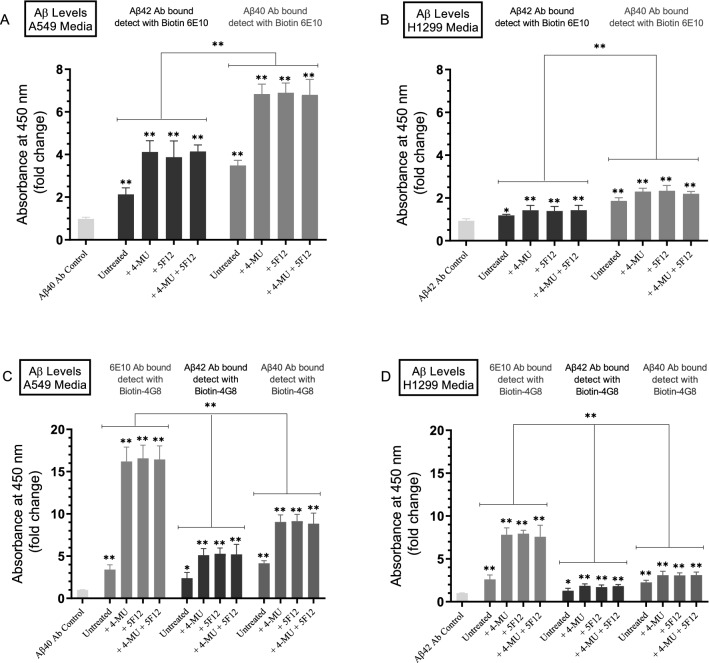
Higher levels of Aβ40 and Aβ42 are found in the media of A549 cells as compared to that of H1299 cells. Cells (0.2 × 10^5^) were grown in 10% FBS-supplemented media for 24 h then incubated in serum-free media for 24 h and treated as indicated for 72 h with 600 µM 4-MU and/or the CD44 antibody (5F12, 5 μg/mL). The media was then collected and the same amount of protein (3 µL of 600 µg/mL total protein) of each sample was used to quantitate Aβ (“Methods” section) using the indicated antibodies. Data from five independent assays, each carried out in triplicate, were quantitated, averaged, normalized, and expressed as fold change relative to controls including all components but using 6E10 antibodies instead of biotin-6E10 (**A**,**B**) or 4G8 antibodies instead of biotin-4G8 (C, D) using the GraphPad 8.4.3 software. The graphs summarize the results expressed as means ± SD (n = 5). Asterisks (*) indicate a statistically significant difference from the corresponding negative control of each cell line, Mann–Whitney test. Statistical differences between different groups were analyzed by an ordinary one-way analysis of variance (ANOVA) followed by Tukey’s post-hoc multiple comparison test. *p < 0.05, **p < 0.01.

The Aβ levels in the conditioned media of control A549 and H1299 cells or those resulting from cell treatment with either 4-MU and/or 5F12, detected using the anti-Aβ (6E10) antibody (Fig. 1), likely represent any Aβ fragment containing the N-terminal 1–16 region. To determine the relative levels of intact Aβ40 and Aβ42 peptides, cells were treated as indicated with 4-MU and/or the CD44 antibody (Fig. 2). The relative levels of Aβ were determined (“Methods” section) using the indicated antibodies and Aβ ELISAs following previously reported protocols^70–72^.

In the absence of 4-MU or 5F12 treatments, the levels of Aβ42 and Aβ40 were ~ 1.8-fold higher in A549 cell media compared to the corresponding values found in the media of H1299 cells (Fig. 2A,B). Compared to untreated samples, treatment of A549 cells with either 4-MU and/or 5F12 resulted in ~ 2.05-fold increase in Aβ42 and Aβ40 levels in the media (Fig. 2A). The same treatments of H1299 cells, however, resulted in ~ 1.15-fold increase in the intact levels of either Aβ42 or Aβ40 (Fig. 2B) in the media relative to untreated controls. These results show that compared to H1299 cell media, higher intact Aβ40 and Aβ42 levels are detected in A549 cell media in cells untreated or treated with either 4-MU and/or 5F12.

Using 6E10 as the capture antibody followed by detection with biotin-4G8 (Fig. 2C,D) showed that Aβ levels in the media of untreated A549 cells were ~ 1.30-fold higher than those found in the media of H1299 cells (Fig. 2C,D). Treatment with either 4-MU and/or 5F12 resulted in ~ 4.85-fold increase in Aβ levels in A549 cell media and a ~ 3.05-fold increase in the media of H1299 cells relative to untreated samples.

Using either Aβ42 or Aβ40 as the capture antibody and biotin-4G8 as the detection antibody, there was ~ 1.9-fold increase in the signal detected by each antibody in A549 cell media relative to that found in the media of H1299 cells in untreated samples. In addition, a comparable fold increase in Aβ levels to that found in Fig. 2A,B was observed relative to untreated samples in A549 cell media (~ 2.20-fold increase) which was higher than that found in H1299 cell media (~ 1.35-fold increase) (Fig. 2C,D).

Collectively, these results show that higher levels of either Aβ40 or Aβ42 are detected in A549 cell media than H1299 cell media without or with cell treatment with 4-MU and/or 5F12. To further investigate possible factors that may account for the differences in intact Aβ40/42 levels in the media of these cell lines, for all subsequent experiments, we selected to focus on measuring Aβ42 and Aβ40 levels using the anti-Aβ42 antibody (reactive to the C-terminus of Aβ42), and anti-Aβ40 antibody (reactive to the C-terminus of Aβ40), as the capture antibodies and biotinylated-anti-Aβ 6E10 as the detection antibody. Moreover, since there was no apparent difference in Aβ levels in the media of either A549 or H1299 cells upon treatment with 4-MU and/or 5F12, only 4-MU treatments relative to untreated cells were examined in the subsequent experiments.

### Treatment of A549 and H1299 cells with the MMP2/9 inhibitor resulted in increased Aβ40 and Aβ42 levels in the media that more closely resembled those found upon cell co-treatment with 4-MU, while treatment with the p53 inhibitor, pifithrin-α, led to decreased Aβ levels in A549 cell media

We next attempted to identify key players involved in the signaling pathway leading to differences in intact Aβ40/42 levels in the media of A549 and H1299 cells, untreated or treated with 4-MU. Treatment with 4-MU has been previously reported to downregulate PI3K and AKT signaling in prostate cancer^32^. Previously in prostate cancer (PCa) cells, 4-MU was found to inhibit HA synthesis and receptor–mediated signaling, while addition of HA to PC3-ML prostate cancer cells blocked 4-MU-mediated downregulation of CD44, pAKT, and PI3K activity^32^. In addition, activation of PI3K/AKT signaling, known to regulate a wide range of cellular processes critical for tumorigenesis including proliferation, survival, and growth^35,37^, was found to attenuate Aβ-induced apoptosis in different cell lines^38,39^. Expression of active AKT resulted in decreased levels of Aβ40 and Aβ42 and less accumulation extracellularly in the conditioned media from Chinese hamster ovary (CHO) cells^73^.

Earlier studies have provided a link between AKT and nuclear factor kappa B (NFκB) in that AKT regulates the transcriptional activity of NFκB^36,74,75^. NFκB activity has also been found essential for PI3K- and AKT-induced oncogenic transformation^74^. Upon stimulation, AKT induced phosphorylation results in NFκB translocation into the nucleus, activating transcription^36,74,75^. Blocking the activity of NFκB was also shown to decrease tumorigenicity^75^.

In lung carcinoma cells, the p53 tumor suppressor was reported to act as a repressor of CD44 protein expression^43^. In cells lacking p53, de-repression of CD44 resulted in increased tumor growth survival, anti-apoptotic and mitogenic effects^43^. Mutations of p53 are common in lung adenocarcinoma^3^ and are known to occur in ~ 34% of NSCLC patients^41,49,76,77^. Recently, p53, known to negatively regulate PI3K gene transcription, was found to suppress EGFR/PI3K/AKT signaling by crosstalk with AKT via feedback loops to regulate the fate of NSCLC cells^76^. AKT was also reported to confer resistance in NSCLC in part by downregulating p53^76^. HA-CD44 signaling is known to be associated with altered activity and expression of p53^78^. Exposure of hepatocarcinoma cells to 4-MU resulted in upregulation of p53^79^ and treatment of human chronic myelogenous leukemia cells, K562, with 4-MU was found to affect apoptosis by increasing p53 expression^80^.

HA binding to CD44 is known to induce the expression and activity of MMPs, involved in degradation of extracellular matrix components and tumor progression^45^. Due to the important role they play in disease pathogenesis such as in AD and cancer, MMP2 and MMP9 gelatinases in particular among the MMPs, are widely considered as highly valuable enzymes^81^. Overexpression of both MMP2 and MMP9 is known to be associated with the progression of different types of cancer including lung cancer and correlates with metastasis and poor prognosis^47,81,82^. Increased HA levels in MCF-10A human mammary epithelial cells, promoted invasiveness by inducing production of MMP2 and MMP9 via the PI3K/AKT pathway^83^. AKT, has been reported to promote the activity of NFκB, known to regulate the transcription of MMP2/9^36,84^. MMP2 and MMP9 are able to degrade both monomeric and fibrillar forms of exogenous Aβ40/42^46,85,86^. Plasmin and MMP9 have been shown to degrade fibrillar Aβ in vitro while other Aβ-degrading enzymes such as neprilysin and insulin-degrading enzyme, mainly have degradative activity towards soluble but not fibrillar forms of Aβ^47,87^. MMP2 and MMP9 were found to degrade Aβ40/42 generating highly soluble, non-fibrillogenic and non-cytotoxic fragments truncated only at the C-terminus ending at positions 16, 30, and 34^85^.

Based on these reports, we hypothesized that differences in the intact levels of Aβ40 and Aβ42 in the media of A549 and H1299 cells as a result of cell treatment with 4-MU might be due to downregulating signaling of PI3K/AKT, NFκB, and MMP2/9 in a p53-dependent manner.

**Figure 3:**
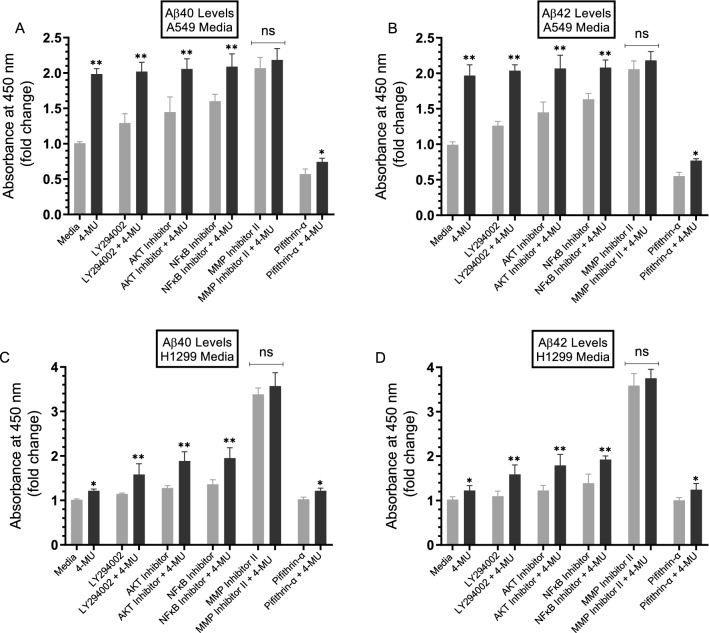
Treatment of cells with the MMP2/9 inhibitor resulted in comparable increases in the levels of Aβ in the media of both A549 and H1299 cells as that upon cell co-treatment with 4-MU, while Aβ levels decreased upon treatment with 4-MU and the p53 inhibitor, pifithrin-α, in A549 cell media only. Cells (0.2 × 10^5^) were grown in 10% FBS-supplemented media for 24 h. The following day, the cell monolayers were incubated in serum-free media for 24 h, then treated as indicated for 72 h with the inhibitors, without and with added 600 µM 4-MU, as described in the “Methods” section. The media was collected then the same amount of protein (3 µL of 600 µg/mL total protein) of each sample was used to quantitate Aβ40/42 (“Methods” section). Data from five independent assays, each carried out in triplicate, were quantitated, averaged, normalized, and expressed as fold change relative to control cells not treated with 4-MU using the GraphPad 8.4.3 software. The graphs summarize the results expressed as means ± SD (n = 5). Asterisks (*) indicate a statistically significant difference from the corresponding cell line control, *p < 0.05, **p < 0.01 of each cell line. Absence of asterisks indicates no significance (ns), Mann–Whitney test.

Cells were treated with inhibitors (Fig. 3) as described in the “Methods” section. Treatment of A549 cells with 4-MU resulted in ~ 2.0-fold increase in Aβ40/42 levels in the media compared to cells not treated with 4-MU (Fig. 3A,B), results consistent with those obtained in Fig. 2A. Levels of Aβ40 and Aβ42 in the media of 4-MU treated H1299 cells increased ~ 1.20-fold (Fig. 3C,D), results close to those obtained in Fig. 2B. In the media of both cell lines, there was an increase in the levels of both Aβ40 and Aβ42 upon cell treatment with the PI3K inhibitor, AKT inhibitor, and NFκB inhibitor. While this increase was not to the same extent as that found upon co-treatment with 4-MU (Fig. 3), these results suggest that these proteins are likely involved in the mechanism regulating the levels of intact Aβ40/42 in the media of A549 and H1299 cells. Treatment with the MMP2/9 inhibitor, however, resulted in the largest increase in the intact levels of either Aβ40 or Aβ42 in the media of both A549 and H1299 cells (Fig. 3). Relative to untreated cells, a larger fold increase in the intact levels of Aβ40 or Aβ42 was observed in the media of H1299 cells treated with the MMP2/9 inhibitor, ~ 3.5-fold, as compared to that observed in the media of A549 cells, ~ 2.0-fold. Relative to untreated A549 cells, treatment with 4-MU and, co-treatment with 4-MU and the MMP2/9 inhibitor, led to a ~ 2.0-fold increase and ~ 2.2-fold increase in the intact levels of Aβ40 or Aβ42, respectively, while the corresponding values obtained in H1299 cell media were ~ 1.2-fold increase and ~ 3.7-fold increase in these levels. These results might be indicative of the presence of more fragmented Aβ40 or Aβ42 in H1299 cell media that become less pronounced by MMP2/9 inhibition. The difference in fold increase in the levels of Aβ40 and Aβ42 in the media of either cell line treated with the MMP2/9 inhibitor without or with 4-MU was not statistically significant suggesting that MMP2/9 may be the main contributors to regulation of intact Aβ levels in the media of these cell lines due to 4-MU treatment.

No change in the levels of either Aβ was found upon treatment of H1299 cells with the p53 inhibitor, pifithrin-α, which is not surprising since they are p53-negative (Fig. 3C,D). Treatment of A549 cells with pifithrin-α, however, resulted in decreased Aβ levels by ~ 40% compared to control (Fig. 3A,B). Moreover, co-treatment of A549 cells with pifithrin-α and 4-MU, led to ~ 1.2-fold increase in the levels of Aβ40 and Aβ42 in the conditioned media relative to cells treated with pifithrin-α alone (Fig. 3A,B). These findings suggest that p53 function can account, in part, for the differences in the intact levels of Aβ40/42 n the media of A549 and H1299 cells.

### Treatment with the p53 inhibitor, pifithrin-α, resulted in higher levels of MMP2 than MMP9 in the conditioned media of A549 cells

CD44-HA interaction is known to induce MMP expression^88,89^. In melanoma cells, treatment with HA was reported to increase secretion and expression of MMP2^89^. Treatment of the human small-cell lung carcinoma cell line, QG90, with HA was shown to strongly activate secretion of MMP2 while expression of antisense CD44s in QG90 cells or pretreatment of cells with the neutralizing anti-CD44 antibody, blocked the HA-dependent secretion of MMP2, highlighting the role of HA-CD44 signaling in HA-dependent MMP2 secretion^90^. Moreover, while HA stimulated MMP2 secretion in QG90 cells, no detection of MMP9 activity was found^90^.

The flavonoid-based synthetic PI3K inhibitor, LY294002, was found to inhibit expression of MMP9 and invasion of glioblastoma (C6) cells^91^. Treatment with LY294002 has been reported to result in antitumorigenic effects, apoptosis, cell growth arrest, and attenuated tumor cell invasion and migration in several tumor models^35^.

Numerous reports have shown that the tumor suppressor gene, *TP53*, has inhibitory effects on cell growth and induces apoptosis when overexpressed in a variety of tumor cells^41,42,49,77,92^. Inactivation of p53 was found to increase lung carcinoma invasion in vitro suggesting that p53 exerts its antineoplastic effects by regulation of cell invasion^93^. Comprehensive proteomic analyses have shown that wild-type p53 alters the secretome, controlling a range of secreted proteins including MMPs^92^. The mechanism by which p53 employs its antitumor effects was found to include downregulating cell invasion and decreased MMP2 protein expression and secreted levels in human melanoma cell conditioned media while no modulation of MMP9 secreted levels was observed^93^. Previously, p53 was reported to modulate expression of MMP2^94^. Both the homeobox transcription factor, HOXA5, and p53 were shown to cooperate to significantly downregulate tumor cell invasion in non-small cell lung cancer, in part by decreasing MMP2 activity^95^. While co-expression of HOXA5 and wild type p53 blocked invasion ability and significantly decreased MMP2 expression level in H1299 cells, no effects were observed on the expression of MMP9^95^.

**Figure 4:**
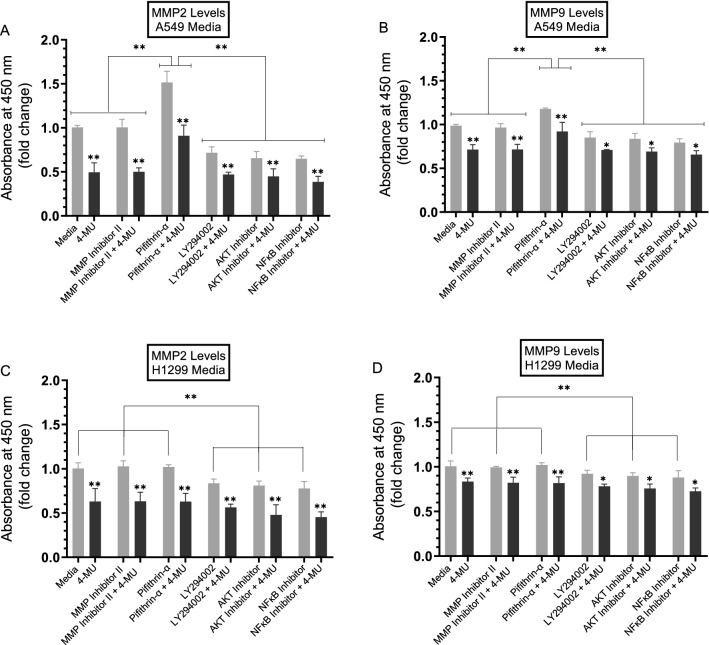
Higher levels of MMP2 than MMP9 were detected in the conditioned media of A549 cells upon treatment with the p53 inhibitor, pifithrin-α. Cells (0.2 × 10^5^) were grown in 10% FBS-supplemented media for 24 h. The following day, the cell monolayers were incubated in serum-free media for 24 h, then treated as indicated for 72 h with the inhibitors without and with 600 µM 4-MU as described in the “Methods” section. The media was collected then the same amount of protein (3 µL of 600 µg/mL total protein) of each sample was used to quantitate MMPs (“Methods” section). Data from five independent assays, each carried out in triplicate, were quantitated, averaged, normalized, and expressed as fold change relative to cells not treated with 4-MU using the GraphPad 8.4.3 software. The graphs summarize the results expressed as means ± SD (n = 5). Asterisks (*) indicate a statistically significant difference from the corresponding samples without 4-MU treatment for each cell line, Mann–Whitney test. Statistical differences between different groups were analyzed by an ordinary one-way analysis of variance (ANOVA) followed by Tukey’s post-hoc multiple comparison test. *p < 0.05, **p < 0.01.

We, therefore, set out to examine the levels of MMP2 and MMP9 in the conditioned media of A549 and H1299 cells treated with the different inhibitors in the absence or presence of 4-MU (Fig. 4) (“Methods” section). Treatment of A549 cells with 4-MU resulted in ~ 2.0-fold decrease in MMP2 levels and ~ 1.3-fold decrease in the levels of MMP9 in the media (Fig. 4A,B). A more modest effect on MMP2 levels (~ 1.5-fold decrease) and MMP9 levels (~ 1.15-fold decrease) in the media was observed upon treatment of H1299 cells with 4-MU (Fig. 4C,D). No change in MMP2/9 levels in the media of either cell line was detected in the presence of the MMP2/9 inhibitor (Fig. 4). Treatment of A549 cells with the p53 inhibitor, pifithrin-α, increased the levels of both MMP2 (~ 1.50-fold increase) and MMP9 (~ 1.15-fold increase) while no effects were observed with this treatment on either MMP2 or MMP9 levels in H1299 cell media (Fig. 4). Since H1299 cells are p53-null, these results indicate that p53 functions to reduce the levels of MMP2/9 in A549 cell media. In the media of both cell lines, treatment of the cells with the PI3K inhibitor, AKT inhibitor, or NFκB inhibitor, the levels of both MMP2 and MMP9 decreased (Fig. 4) suggesting that these proteins are likely involved in the mechanism regulating the levels of MMP2/9 in the media of A549 and H1299 cells.

### Treatment of A549 cells with the p53 inhibitor, pifithrin-α, resulted in upregulation of PI3K and AKT activities

The PI3K/AKT pathway is known to act via a range of substrates to promote metastasis and proliferation^35^. Interactions between the PI3K/AKT and p53 pathways are known to occur through different mechanisms that include regulation of the PI3K/AKT pathway by p53 via activation of the PTEN tumor suppressor^96^. It was previously reported that UV exposure of A549 cells resulted in increased expression of p53 and decreased levels of PI3K p110α and phosphorylated AKT^97^.

**Figure 5:**
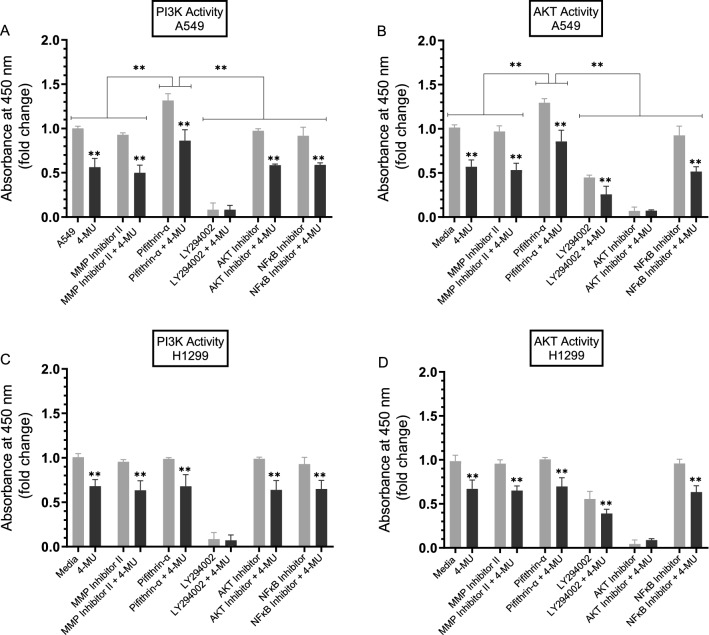
The activity of either PI3K or AKT is upregulated upon treatment of A549 cells with the p53 inhibitor, pifithrin-α. Cells (0.2 × 10^5^) were grown in 10% FBS-supplemented media for 24 h. The following day, the cell monolayers were incubated in serum-free media for 24 h, then treated as indicated for 72 h with the inhibitors without or with 600 µM 4-MU. The PI3K activity was assayed by the Total In-Cell ELISA Kit and the AKT activity was measured on the same amount of protein (3 µL of 600 µg/mL total protein) of cell lysate as described in the “Methods” section. Data from five independent assays, each carried out in triplicate, were quantitated, averaged, normalized, and expressed as fold change relative to cells not treated with 4-MU using the GraphPad 8.4.3 software. The graphs summarize the results expressed as means ± SD (n = 5). Asterisks (*) indicate a statistically significant difference from the corresponding samples without 4-MU treatment for each cell line, Mann–Whitney test, while the absence of asterisks indicates no significance. Statistical differences between different groups were analyzed by an ordinary one-way analysis of variance (ANOVA) followed by Tukey’s post-hoc multiple comparison test. *p < 0.05, **p < 0.01.

To examine the effect of the inhibitors on the activity of PI3K and AKT under our conditions, cells were treated as indicated with the inhibitors, then the PI3K and AKT assays were carried out as described in the “Methods” section. The activities of PI3K and AKT were decreased by ~ 1.8-fold and ~ 1.5-fold upon treatment of A549 and H1299 cells with 4-MU, respectively (Fig. 5). The activities of PI3K and AKT increased ~ 1.35-fold (Fig. 5A,B) upon A549 cell treatment with the p53 inhibitor, pifithrin-α. Not surprisingly, no effects were found upon treatment of H1299 cells with pifithrin-α (Fig. 5C,D) since they are p53-negative^49^. These results suggest that p53 functions to downregulate PI3K and AKT activities in A549 cells.

### Treatment of A549 cells with the p53 inhibitor, pifithrin-α, resulted in increased phospho/total NFκB ratio

Multiple phosphorylation sites on the p65 subunit of NFκB have been mapped in both the N-terminal Rel homology domain as well as the C-terminal transactivation domain^98^.

Phosphorylation of the p65 subunit of NFκB on S536 in the C-terminal transactivation domain is associated with translocation of NFκB subunits and enhancement of NFκB transactivation, while reduced S536 phosphorylation results in decreased NFκB activity and cell growth^99^.

**Figure 6:**
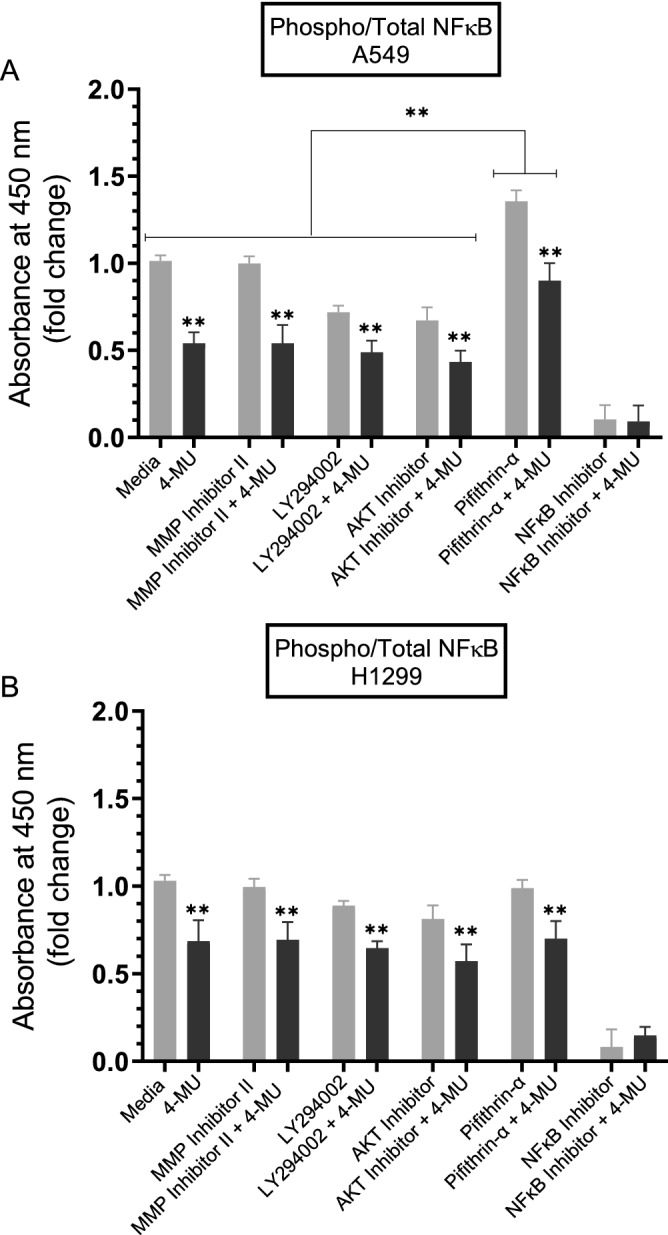
Phospho/Total NFκB ratio is increased upon treatment of A549 cells with the p53 inhibitor, pifithrin-α. Cells (0.2 × 10^5^) were grown in 10% FBS-supplemented media for 24 h. The following day, the cell monolayers were incubated in serum-free media for 24 h, then treated as indicated for 72 h with the inhibitors without and with 600 µM 4-MU as described in the “Methods” section. The cell lysate was prepared and the same amount of protein (600 µg/mL total protein) of each sample was used to assay for NFκB activity as described in the “Methods” section. Data from five independent assays, each performed in triplicate, were quantitated, averaged, normalized, and expressed as fold change relative to cells not treated with 4-MU using the GraphPad 8.4.3 software. The graphs summarize the results expressed as means ± SD (n = 5). Asterisks (*) indicate a statistically significant difference from the corresponding samples without 4-MU treatment for each cell line, Mann–Whitney test, while the absence of asterisks indicates no significance. Statistical differences between different groups were analyzed by an ordinary one-way analysis of variance (ANOVA) followed by Tukey’s post-hoc multiple comparison test. **p < 0.01.

To examine the effect of the inhibitors on the ratio of phospho/total NFκB, cells were treated as indicated with the inhibitors followed by assaying the NFκB activity as described in the “Methods” section. Treatment of A549 and H1299 cells with 4-MU resulted in ~ 2.0-fold and ~ 1.4-fold decrease in NFκB phosphorylation, respectively (Fig. 6). While no apparent effects were observed upon adding the MMP2/9 inhibitor, treatment of A549 cells with LY294002 resulted in ~ 1.3-fold decrease in phosphorylation while this decrease was ~ 1.5-fold when A549 cells were treated with the AKT inhibitor (Fig. 6A). While the trends were similar, the effects were more modest when treating H1299 cells with LY294002 and the AKT inhibitor resulting in a decrease of ~ 1.18-fold and ~ 1.25-fold, respectively, in NFκB phosphorylation (Fig. 6B). Treatment of A549 cells with the p53 inhibitor, pifithrin-α, increased NFκB phosphorylation by ~ 1.4-fold (Fig. 6A) while no effects were observed when using H1299 cells (Fig. 6B). The lack of effect upon treatment of H1299 cells with pifithrin-α is not surprising since they are p53-null. The increase in NFκB phosphorylation by inhibiting p53 suggests that p53 is an antagonist of NFκB phosphorylation in A549 cells. These results are supportive of previous reports indicating that NFκB and p53 have opposing effects in cancer cells with antagonistic signaling cross-talk^100^. Both p53 and NFκB have been previously shown to cross-regulate each other’s activity and suppress each other’s ability to enhance gene expression^100^. Increased NFκB activity has been reported in p53-null mice and loss of p53 was shown to trigger activation of NFκB in a mouse model of KrasG12D-driven lung adenocarcinoma while restoring p53 in p53-null lung tumors led to inhibition of NFκB and tumor suppression^92,101^.

### Comparable increases in the levels of Aβ were observed in media of A549 and H1299 cells treated with either MMP2 siRNA or in combination with 4-MU

**Figure 7:**
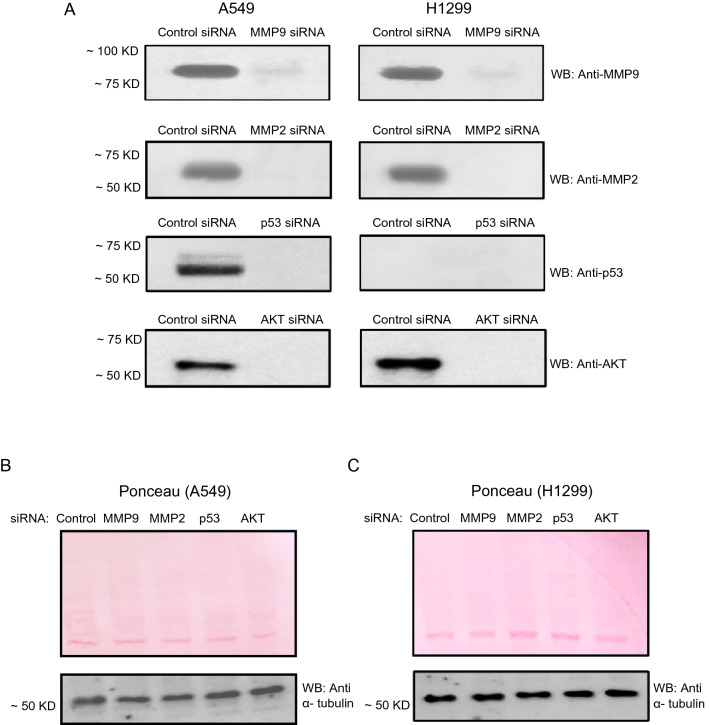
Knockdown of MMP9, MMP2, p53, and AKT by siRNA transfection. Cells were seeded at a density of 2 × 10^4^ cells in 25 cm^2^ flasks. The following day, siRNA targeted against the indicated proteins was mixed with Lipofectamine 2000 transfection reagent (ThermoFisher) for 20 min at RT. The mixtures were then added to the cells to a final concentration of 100 nM for each siRNA. The cells were then allowed to incubate at 37 °C in serum-free media for 72 h. The same concentration of total protein (15 µL of 600 µg/mL) of the cell lysates (**A**) was used for Western blotting using the indicated antibodies. Total protein (Ponceau staining) and α-tubulin (**B**,**C**) served as loading controls.

**Figure 8:**
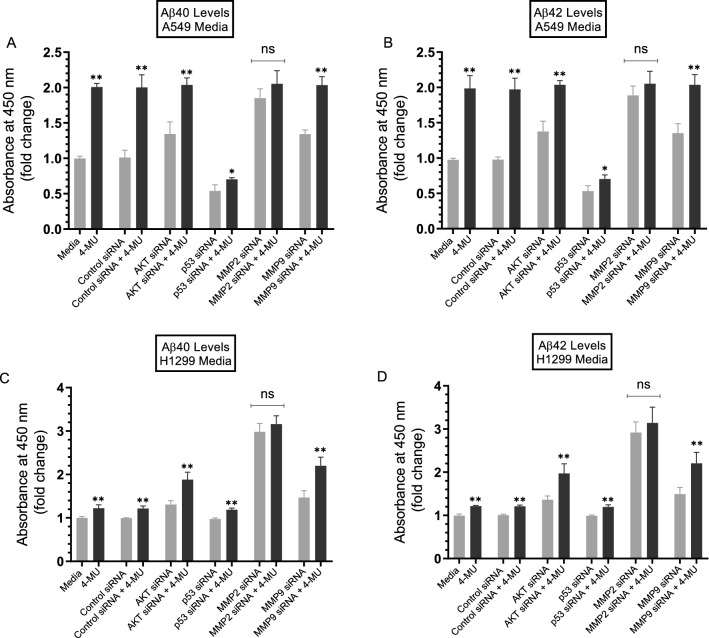
Treatment of cells with MMP2 siRNA resulted in comparable increases in the levels of Aβ in the media of both A549 and H1299 cells as that upon cell co-treatment with 4-MU. Cells (0.2 × 10^5^) were grown in 10% FBS-supplemented media for 24 h. The following day, the cell monolayers were incubated in serum-free media for 24 h, then treated for 72 h with the indicated siRNAs without or with added 600 µM 4-MU as described in the “Methods” section. The same amount of protein (3 µL of 600 µg/mL total protein) of the media was used to quantitate Aβ (“Methods” section). Data from five independent assays, each carried out in triplicate, were quantitated, averaged, normalized, and expressed as fold change relative to cells not treated with 4-MU using the GraphPad 8.4.3 software. The graphs summarize the results expressed as means ± SD (n = 5). Asterisks (*) indicate a statistically significant difference from the corresponding samples without 4-MU treatment, *p < 0.05, **p < 0.01 of each cell line. Absence of asterisks indicates no significance (ns), Mann–Whitney test.

When compared with other inhibitors (Fig. 3), our data suggest that inhibition of MMP2/9 led to increased levels of Aβ40/42 in the media of A549 and H1299 cells that more closely resembled those observed upon co-treatment with 4-MU. Moreover, p53 appears to be an important regulator of Aβ40/42 levels since inhibiting its function with pifithrin-α, in the absence or presence of 4-MU, resulted in decreased intact levels of Aβ40/42 in A549 cell media (Fig. 3). Therefore, we next set out to examine the relative contributions of either MMP2 or MMP9 to intact Aβ levels by treatment with siRNA targeted against either MMP (“Methods” section) (Figs. 7, 8). Moreover, to further verify the involvement of p53 in regulating Aβ levels in the conditioned media, we tested the effects of treating the cells with p53 siRNA on Aβ levels (Figs. 7, 8).

Treatment of cells with AKT siRNA (Figs. 7, 8) resulted in increased Aβ levels in the media of both A549 and H1299 cells with the most pronounced effect (~ 1.45-fold increase) found in A549 cell media (Fig. 8A,B). These results are comparable to those obtained using the AKT inhibitor (Fig. 3). Treatment of A549 cells with p53 siRNA led to ~ 1.8-fold decrease in the intact levels of Aβ relative to cells transfected with control siRNA (Fig. 8A,B). Similarly, A549 cells transfected with p53 siRNA and treated with 4-MU resulted in ~ 2.85-fold decrease in the levels of intact Aβ relative to cells treated with control siRNA and 4-MU (Fig. 8A,B). This decrease was comparable to that observed upon inhibition of p53 using pifithrin-α (Fig. 3A,B) suggesting that the function of p53 is important for regulating the levels of intact Aβ in A549 cell media. No effects were found on the levels of either Aβ in H1299 cells transfected with p53 siRNA (Fig. 8C,D), results consistent with the lack of p53 in H1299 cells. In both cell lines, treatment with MMP2 or MMP9 siRNAs resulted in increased intact levels of Aβ40/42 in the media, however, Aβ levels closely mimicking those measured by co-treatment with 4-MU, were observed upon using MMP2 siRNA (Fig. 8). These results clearly highlight the importance of MMP2/9 in regulating intact Aβ40/42 levels in lung cancer cell media in the absence or presence of 4-MU and might point to MMP2 out of the two MMPs as the main regulator of these levels.

### MMP2/9 siRNA treatment of either A549 or H1299 cells resulted in decreased cell viability and increased apoptosis, an effect diminished upon the same treatment with Aβ immunodepleted media

**Figure 9:**
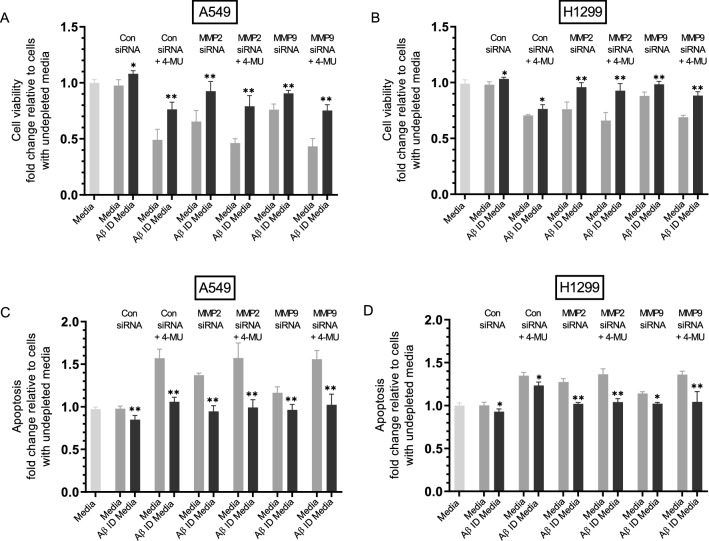
Treatment of either A549 or H1299 cells with MMP2/9 siRNA decreased cell viability and increased apoptosis, an effect reduced upon treatment with Aβ immunodepleted (ID) media. Aβ immunodepleted media (ID) was prepared by growing cells (0.2 × 10^5^) in 10% FBS-supplemented media for 24 h. The cells were then incubated in serum-free media overnight then with the indicated treatments for 72 h. The media was then collected and depleted from Aβ sequentially using 6E10, Aβ40 and Aβ42 antibodies (“Methods” section). Viability and apoptosis of A549 and H1299 cells were assessed as described in the “Methods” section. Briefly, cells were seeded in 96-well plates at 0.2 × 10^5^ cells per well in 10% FBS-supplemented media. The next day, the cell monolayers were incubated in serum-free media for 12 h, then treated with 300 μL of the control and Aβ ID media (0.5 μg/μL) for 72 h with the media containing the specific components in the various treatments replaced every 12 h. Data were processed using the GraphPad 8.4.3 software. The graphs summarize the results expressed as means ± SD (n = 3) of three separate experiments, each performed in triplicate. Asterisks (*) indicate a statistically significant difference between each treatment relative to non-depleted samples, Mann–Whitney test, *p < 0.05, **p < 0.01.

Our results (Fig. 8) show that MMP2 siRNA transfection of either A549 or H1299 cells resulted in increased intact Aβ levels that are comparable to those obtained by co-treatment with 4-MU. We next asked whether the resulting increased levels of Aβ had an effect on cell viability or apoptosis (Fig. 9). To address this question, media was first collected from different cell treatments then immunodepleted (ID) of Aβ (“Methods” section). Next, viability and apoptosis of A549 and H1299 cells were assessed as described in the “Methods” section and in Fig. 9 legend.

Immunodepletion of Aβ from media of either A549 or H1299 cells transfected with control siRNA resulted in increased viability and decreased apoptosis (Fig. 9) suggesting that Aβ has cytotoxic functions in the media of these cell lines. Incubation of A549 cells with media from control siRNA transfections treated with 4-MU decreased cell viability by ~ 2.0-fold (Fig. 9A), however, when treated with media ID of Aβ under the same conditions, there was a ~ 1.55-fold relative increase in cell viability. Conversely, relative to cells treated with media from control siRNA transfections, apoptosis increased ~ 1.55-fold upon treatment of A549 cells with media co-treated with control siRNA and 4-MU, a value that decreased by ~ 1.48-fold when Aβ was immunodepleted (Fig. 9C). Treatment of A549 cells with MMP2 siRNA media decreased cell viability and increased apoptosis by ~ 1.35-fold (Fig. 9A,C). Immunodepletion of Aβ from media of A549 cells treated with MMP2 siRNA resulted in ~ 1.42-fold increase in viability (Fig. 9A) and ~ 1.45-fold decrease in apoptosis (Fig. 9C) compared to undepleted media. Treatment of A549 cells with media from MMP9 siRNA transfection resulted in ~ 1.30-fold decrease in A549 cell viability and ~ 1.20-fold increase in apoptosis. Immunodepletion of Aβ from MMP9 siRNA transfected cell media resulted in ~ 1.18-fold increase in A549 cell viability and ~ 1.16-fold decrease in apoptosis (Fig. 9A,C), effects less pronounced than those observed for MMP2 transfections. Relative to media of cells transfected with control siRNA, incubation of A549 cells with media from co-treatment with MMP2 siRNA and 4-MU or with MMP9 siRNA and 4-MU decreased cell viability ~ 2.0-fold and increased apoptosis ~ 1.55-fold while depletion of Aβ under these conditions led to a relative increase in cell viability and decrease in apoptosis by ~ 1.50-fold (Fig. 9A,C), effects comparable to those found with A549 cells incubated with media co-treated with control siRNA and 4-MU.

Treatment of H1299 cells with media from control siRNA transfection in the presence of 4-MU resulted in ~ 1.40-fold decrease in cell viability (Fig. 9B) and ~ 1.35-fold increase in apoptosis (Fig. 9D) while the same treatment after ID of Aβ resulted in only ~ 1.10-fold increase in viability or decrease in apoptosis relative to cell treatment with undepleted media. H1299 cells treated with media ID of Aβ after transfection with MMP2 siRNA led to ~ 1.30-fold increase in cell viability and decrease in apoptosis relative to treatment with undepleted media. H1299 cell incubation with media ID of Aβ after co-treatment with MMP2 siRNA and 4-MU resulted in ~ 1.40-fold increase in cell viability and ~ 1.35-fold decrease in apoptosis compared to undepleted media (Fig. 9B,D). The effects were more modest using media from MMP9 siRNA transfections. There was ~ 1.15-fold increase in viability and decrease in apoptosis upon cell treatment with Aβ ID media of H1299 cells transfected with MMP9 siRNA relative to undepleted media, while the same treatments in the presence of 4-MU led to ~ 1.30-fold increase in viability and decrease in apoptosis (Fig. 9B,D).

These results suggest that treatment with MMP2 siRNA leads to a greater increase in Aβ cytotoxicity compared to that found with MMP9 siRNA treatment. This observation might also suggest that MMP9 is not the predominant regulator of Aβ cytotoxic functions in the media of these cell lines. Moreover, these results might correlate with the findings (Fig. 8) showing the relatively higher levels of intact Aβ in the media upon treatment with MMP2 siRNA as compared to MMP9 siRNA treatment, and that these higher levels more closely correlate with those observed by co-treatment with 4- MU. While both human MMP2 and MMP9 were found to degrade Aβ generating nontoxic soluble fragments, MMP9 was reported to be less efficient^85^. Taken together, our results suggest that cell treatment with MMP2 siRNA is largely responsible for the increased intact levels of Aβ (Fig. 8) resulting in Aβ cytotoxicity (Fig. 9).

## Discussion

Recently, a rapidly growing body of scientific evidence has steadily emerged showing a reciprocal association between AD and cancer, in that AD patients exhibit some protection against certain types of cancer, and decreased risk of developing cancer with time^102^. Conversely, individuals with cancer diagnosis might be less likely to subsequently live long enough to develop AD^103–108^. In glioblastoma and in other types of cancers including lung cancer, the incidence of AD was found to be decreased^108^. Current evidence suggests that Aβ could inhibit tumor cell growth and is protective against certain types of cancer^68,109^. Proliferation of human breast adenocarcinoma, melanoma, and glioblastoma^68^ was inhibited following addition of conditioned media containing Aβ to cancer cell lines. Tumor growth in mice was suppressed upon direct injection of Aβ into human lung adenocarcinoma xenografts^109^.

In this study, we set out to better understand mechanisms regulating Aβ levels in A549 (p53 wild-type) and H1299 (p53-null) cell media. In untreated controls, Aβ levels were found to be comparable in the media of both cell lines (Fig. 1) using anti-Aβ specific antibodies (6E10), known to react with monomers, oligomers, and fibrils of Aβ^54,55^ and recognize the N-terminal hydrophilic Aβ sequence, amino acids 1–16, an epitope exposed in Aβ aggregates^54^. Higher levels of Aβ, however, were detected in the media of A549 and H1299 cells upon treatment with either 4-MU and/or 5F12 (Fig. 1). This increase in Aβ levels might result from disruption of HA signaling, since 4-MU is thought to exert its effects primarily due to inhibition of HA synthesis^31–34^ and because the anti-CD44 antibody (5F12) is known to be antagonistic towards HA-CD44 molecular interactions^58,59^. The increase in Aβ levels was comparable in the media of both A549 (p53 wild-type) and H1299 (p53-null) cells suggesting that p53 does not play a role in this process.

Detection of Aβ levels in the conditioned media of A549 and H1299 cells without or with treatment with 4-MU and/or 5F12 using the anti-Aβ 6E10 antibody (Fig. 1), is most likely a representation of any Aβ fragment containing the N-terminal 1–16 region which might include Aβ1-x, Aβ1-42 or Aβ1-40. In order to determine the relative levels of different Aβ fragments, Aβ40 and Aβ42 were measured by two-site binding ELISAs as described in the “Methods” section (Fig. 2). In untreated cells, the levels of both Aβ42 and Aβ40 were higher in the media of A549 cells relative to those in H1299 cell media (Fig. 2A,B). Relative to untreated cells, treatment of A549 cells with 4-MU and/or 5F12 resulted in a comparable fold increase in the levels of both Aβ42 and Aβ40 (~ 2.05-fold) as compared to a more modest increase in those levels in H1299 cell media (~ 1.15-fold, Fig. 2) under the same conditions. The results obtained by using the anti-Aβ 6E10 antibodies that recognize an epitope in the N-terminal region of both Aβ40 and Aβ42 (Fig. 1), show that the levels of Aβ are comparable in the media of both A549 and H1299 cells, untreated or treated with 4-MU and/or 5F12. The results obtained by measuring the intact levels of Aβ40 and Aβ42, however, show that there is a higher level of intact Aβ40 and Aβ42 in the media of A549 cells relative to that of H1299 cell media, suggesting the possible existence of more proteolytic degradation of Aβ40 and Aβ42 in the media of H1299 cells (Fig. 2).

**Figure 10:**
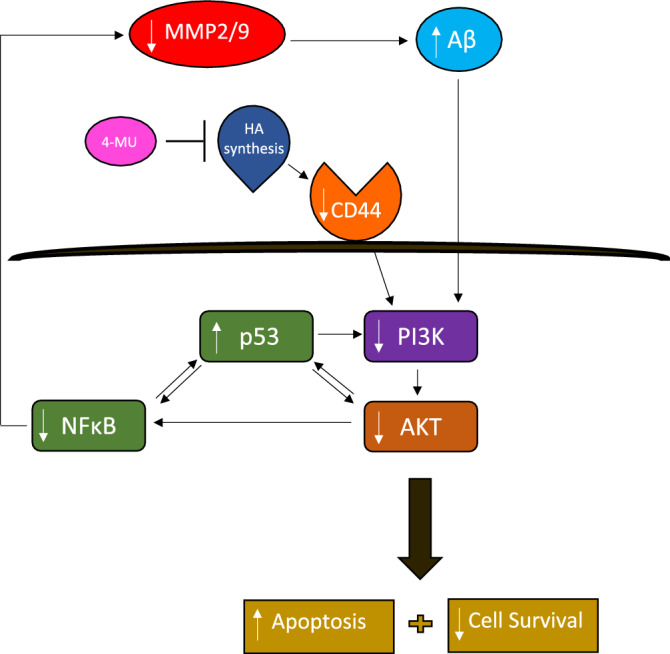
Representation of the main hypothesis and findings of this study.

We next attempted to identify key molecular players involved in regulating the levels of intact Aβ40/42 in the media of A549 and H1299 cells, untreated or treated with 4-MU (Figs. 3, 10). Interaction of HA with CD44 is known to activate MMP signaling promoting tumor progression^20,26,45^. Treatment with 4-MU has been previously reported to downregulate PI3K and AKT signaling in prostate cancer^32^. Moreover, 4-MU likely disrupts HA signaling pathways, downregulating AKT and downstream effectors, including MMP2 and MMP9^110^ since HA addition was shown to prevent the 4-MU induced down regulation of CD44, MMP2 and MMP9 at the protein and mRNA levels^111^*.* In addition, 4-MU was found to result in downregulation of P-AKT and NFκB reporter activity^111^, an activity essential for PI3K- and AKT-induced oncogenic transformation^74^. In MCF-10A human mammary epithelial cells, increased levels of HA promoted invasiveness by increasing production of MMP2 and MMP9 via the PI3K/AKT pathway^83^. AKT, was shown to promote the activity of NFκB, known to regulate the transcription of MMP2/9^36,84^. MMP2 and MMP9 overexpression, and links to the progression of a wide range of cancers, have been well-documented and due to their involvement in the pathophysiology of disease, MMP2 and MMP9 are generally considered as the most important enzymes among the MMPs^47,81,84^. Increased levels of both MMP2 and MMP9 have also been detected in the serum of patients with AD^81,86,87^. Cytoplasmic and stromal MMP2 expression was reported in lung cancer patients and many studies have correlated lung cancer invasion and metastasis with high expression of MMP2 and MMP9^112,113^. Moreover, adenovirus-mediated knockdown of MMP2 inhibited tumor growth and blocked formation of lung nodules^114^.

Known to negatively regulate PI3K gene transcription, p53 was recently found to suppress EGFR/PI3K/AKT signaling by crosstalk with AKT via feedback loops to regulate the fate of NSCLC cells^76^. AKT was also shown to increase resistance in NSCLC in part via p53 downregulation^76^. HA-CD44 signaling is also known to be associated with altered activity and expression of p53^78^. Treatment of hepatocarcinoma cells with 4-MU led to upregulation of p53^79^ and treatment of human chronic myelogenous leukemia cells, K562, with 4-MU was reported to affect apoptosis by increasing expression of p53^80^. Using inhibitors targeted towards key proteins suspected to be involved in the mechanism of action of 4-MU in this study (Fig. 10), we found that PI3K, AKT, and NFκB are likely involved in the mechanism regulating the intact levels of Aβ40/42 in the media of A549 and H1299 cells (Figs. 3, 10). However, increased intact levels of Aβ40 and Aβ42 in A549 and H1299 cell media that more closely resembled those found upon cell co-treatment with 4-MU, resulted from using the MMP2/9 inhibitor (Fig. 3) suggesting that MMP2/9 may be the main contributors to regulation of intact Aβ40/42 levels in the media. Relative to cells untreated or treated with 4-MU, our results also show that treatment with the p53 inhibitor, pifithrin-α, led to decreased Aβ40/42 levels in A549 cell media (Fig. 3A,B) while no change in these levels was found in the media of H1299 cells under the same conditions, findings that are not surprising since H1299 cells are p53-negative (Fig. 3C,D). Differences in the levels of intact Aβ40/42 might, therefore, be accounted for in part by p53 function.

Treatment with HA was reported to increase expression and secretion of MMP2 in melanoma cells^89^. Treatment of the human small-cell lung carcinoma cell line, QG90, with HA was reported to strongly promote MMP2 secretion while expression of antisense CD44s in QG90 cells or pretreatment of cells with the neutralizing anti-CD44 antibody, blocked the HA-dependent secretion of MMP2, highlighting the important role of HA-CD44 signaling in HA-dependent MMP2 secretion^90^. In addition, while HA stimulated secretion of MMP2 in QG90 cells, no MMP9 activity was detected^90^. The mechanism by which p53 employs its antitumor effects was reported to include downregulating cell invasion and reduced MMP2 protein expression and secreted levels in human melanoma cell conditioned media, while no modulation of MMP9 secreted levels was found^93^. Previous reports have shown that p53 modulates the expression of MMP2^94^ and both p53 and the homeobox transcription factor, HOXA5, were shown to cooperate, significantly downregulating tumor cell invasion in non-small cell lung cancer, in part by decreasing MMP2 activity^95^. Furthermore, while co-expression of HOXA5 and wild type p53 decreased invasion and significantly inhibited MMP2 expression level in H1299 cells, no effects were observed on MMP9 expression^95^. Our results show that treatment with 4-MU negatively regulated the levels of MMP2 in the media of both cell lines and to a larger extent than MMP9 levels (Fig. 4). Both MMP2 and MMP9 levels decreased (Fig. 4) in the media of both A549 and H1299 cells as a result of cell treatment with inhibitors targeting PI3K, AKT, and NFκB suggesting that these proteins are likely involved in the mechanism regulating the levels of MMP2/9 in the media of A549 and H1299 cells. While no effects were observed in H1299 cell media, treatment with pifithrin-α resulted in higher levels of MMP2 than MMP9 in the conditioned media of A549 cells (Fig. 4). Since H1299 cells are p53-null, these results indicate that p53 functions to reduce the levels of MMP2/9 in A549 cell media, albeit to different extents.

Interactions between the p53 and PI3K/AKT pathways are known to occur through different mechanisms that include p53 regulation of the PI3K/AKT pathway via activation of the PTEN tumor suppressor^96^. UV exposure of A549 cells led to increased p53 expression and decreased levels of PI3K p110α and phosphorylated AKT^97^. Treatment of A549 cells with the p53 inhibitor, pifithrin-α, resulted in upregulation of PI3K and AKT activities (Fig. 5A,B) while no effects were observed with the same treatment of H1299 cells (Fig. 5C,D), suggesting that p53 functions to downregulate PI3K and AKT activities in A549 cells. We also found that treatment of A549 cells with pifithrin-α resulted in increased phospho/total NFκB ratio (Fig. 6) suggesting that p53 is an antagonist of NFκB phosphorylation in A549 cells. These results support previous findings showing that NFκB and p53 have opposing effects in cancer cells with antagonistic signaling cross-talk^100^. NFκB activity was found to increase in p53-null mice and p53 loss triggered activation of NFκB in a mouse model of KrasG12D-driven lung adenocarcinoma while restoring p53 in p53-null lung tumors blocked NFκB and suppressed tumor growth^92,101^.

Using siRNA targeted against MMP2 and MMP9, we found increased intact levels of Aβ40 and Aβ42 in the media of A549 and H1299 cells (Figs. 7, 8). However, Aβ levels closely mimicking those observed upon co-treatment with 4-MU were found by cell transfection with MMP2 siRNA (Fig. 8). These findings highlight the importance of MMP2/9 in regulating the intact levels of Aβ40/42 in the conditioned media of lung cancer cells and possibly point to MMP2, among the two MMPs, as the main regulator of those levels. Knockdown of p53 in A549 cells resulted in decreased intact levels of both Aβ40 and Aβ42 in the media (Fig. 8A,B). This observation might be linked to the increased levels of MMP2/9 (Fig. 4), PI3/AKT activities (Fig. 5), and the phospho/total NFκB ratio (Fig. 6) upon treatment of A549 cells with the p53 inhibitor, pifithrin-α.

We also subjected the cells to different treatments (Fig. 9) and used media immunodepleted of Aβ from these treatments to examine the effects of Aβ on cell viability or apoptosis. Our results show that incubation of either A549 or H1299 cells with media obtained from MMP2 siRNA transfections resulted in decreased cell viability and increased apoptosis, an effect that was less pronounced upon the same treatment with Aβ immunodepleted media. The effects due to Aβ on either viability or apoptosis of A549 and H1299 cells upon treatment with MMP9 siRNA were less pronounced (Fig. 9) suggesting that, of the two MMPs, MMP2 is the predominant regulator of Aβ cytotoxic functions in these cells. These findings might be explained, in part, by previously published reports showing that human MMP2 and MMP9 can degrade Aβ generating nontoxic soluble fragments with MMP9 being the least efficient^85^.

While our findings do not explain the mechanisms behind the overall increased levels of Aβ detected upon using anti-Aβ 6E10 antibodies (Fig. 1), they do point to the involvement of HA-CD44 signaling in this process and indicate that these mechanisms operate independently of p53 since the levels were comparable in the media of both A549 (p53-positive) and H1299 (p53-negative) cells. While p53 does not appear to regulate the overall levels of Aβ detected using the anti-Aβ 6E10 antibodies (Fig. 1), it does appear to play a role in regulating the intact Aβ40/42 levels in the media. Our results show that the levels of intact Aβ40/42 are higher in the media of A549 cells than those in the media of H1299 cells (Fig. 2). These higher intact levels in A549 cell media decreased upon cell treatment with either the p53 inhibitor, pifithrin-α (Fig. 3), or upon transfection with p53 siRNA (Fig. 8). While it is expected that multiple mechanisms act cooperatively to regulate the steady-state levels of intact Aβ40/42 in the conditioned media, our data suggest that cell treatment with MMP2 siRNA is more effective than that using MMP9 siRNA in increasing the levels of intact Aβ40/42 (Fig. 8) resulting in Aβ cytotoxicity (Fig. 9). One likely mechanism by which Aβ40/42 can exert the cytotoxic effects is via downregulation of PI3K/AKT signaling (Fig. 10). Aβ is known to inhibit PI3K activity and was previously reported to interrupt PI3K-AKT-mTOR signaling in rat cortical neurons^35,40,115^. Aβ resulted in inhibition of the PI3K pathway in neuronal cells, inducing neurotoxicity, while activation of PI3K signaling with a direct PI3K activator resulted in neuroprotection in Aβ-induced neuronal cell death^40^. In addition, activation of PI3K/AKT signaling was shown to block Aβ-induced apoptosis^38,39^. Research into the mechanisms behind the increased levels of Aβ in the media due to cell treatment with either 4-MU and/or 5F12, and investigation of whether Aβ40 and/or Aβ42 induce cytotoxic effects in A549 and H1299 cells via downregulation of the PI3K/AKT cascade is currently ongoing in our laboratory.

## Methods

### Materials

Most of the material used in this study was purchased as we reported earlier^50–52,57^. Phosphate Buffered Saline (PBS), nitrocellulose membranes, 4-Methylumbelliferone (4-MU, M1381), streptavidin–horseradish peroxidase (HRP) conjugate, Ponceau S solution, LY294002 hydrochloride, hydrogen peroxide solution, AKT Inhibitor (Calbiochem), pifithrin-α p-Nitro, were purchased from Sigma-Aldrich. MMP2/MMP9 Inhibitor II was purchased from EMD Millipore. CD44 antibody (5F12) (MA5-12394), mouse IgG isotype control, (mIgG), α-tubulin monoclonal antibody (DM1A), 3,3′,5,5′-tetramethylbenzidine (TMB), and lipofectamine 2000 transfection reagent were from ThermoFisher. RealTime-Glo annexin V apoptosis and necrosis assay kit (JA1000) was purchased from Promega. Rabbit anti-goat IgG (HRP) (ab6741) and donkey anti-mouse IgG (HRP) (ab205724) were purchased from Abcam. Monoclonal amyloid-β antibody (sc-53822), goat anti-rabbit IgG-HRP (sc-2004), NFκB inhibitor (CAS 213546-53-3), MMP2 siRNA (sc-29398), MMP2 antibody (sc-13594), MMP9 siRNA (sc-29400), MMP9 antibody (sc-393859), and m-IgGκ BP-HRP, were from Santa Cruz Biotechnology. The BCA protein assay kit and the super signal west pico luminol (chemiluminescence) reagent were from Pierce. SignalSilence p53 siRNA I (6231), SignalSilence AKT siRNA, SignalSilence Control siRNA (Unconjugated, 6568), p53 antibody (9282), and the Akt Antibody (9272) were purchased from Cell Signaling Technology. Anti-Aβ (6E10, 1–16) antibody, anti-Aβ (4G8, 17–24) antibody, anti-Aβ42 antibody that is reactive to the C-terminus of Aβ42, anti-Aβ40 antibody that is reactive to the C-terminus of Aβ40, biotin anti-Aβ (4G8, 17–24) antibody, and biotin anti-Aβ (6E10, 1–16) antibody were from BioLegend. No animals were involved in the study.

### Cell culture

Human NSCLC cell lines, A549 (ATCC CCL-185) and H1299 (ATCC CRL-5803), were purchased from the American Type Culture Collection (ATCC, Manassas, VA). Cells were seeded as we reported earlier^50,51,57^ in 5 mL HyClone Dulbecco’s modified Eagle’s media/nutrient mixture F-12 (DMEM/F12) (GE Healthcare Life Sciences, Pittsburgh, PA), supplemented with 10% Fetalgro bovine growth serum (FBS, RMBIO, Missoula, MT), 50 U/mL penicillin, and 50 U/mL streptomycin (Invitrogen Life Technologies, Carlsbad, CA) in 25 cm^2^ tissue culture flasks, and allowed to grow overnight in an incubator at 37 °C, 95% humidity, and 5% CO_2_. The cells were counted after trypan blue staining, with a hemocytometer.

When inhibitors were used, cells were treated with inhibitors targeted against PI3K (LY294002, 14.5 μM), AKT (AKT inhibitor, 1.75 μM), p53 (pifithrin-α, 10 μM), NFκB (NFκB inhibitor, 18 μM), MMP2/9 (MMP inhibitor II, 5 μM) as indicated.

### ELISA

ELISAs were conducted as we reported previously^50,51,116^. Nunc MaxiSorp 96-well Flat Bottom plate (ThermoFisher) wells were coated with samples as indicated. The plates were incubated overnight at 4 °C on a shaker to allow binding to the plate wells. After the incubation, the wells were washed 4 × with TBST, filled with 400 µL blocking buffer (110 mM KCl, 5 mM NaHCO_3_, 5 mM MgCl_2_, 1 mM EGTA, 0.1 mM CaCl_2_, 20 mM HEPES, 1% BSA, pH 7.4), and incubated overnight at 4 °C on a shaker. The wells were then washed 4 × with TBST and 100 µL of sample at the desired concentration, was added to each well followed by incubation overnight at 4 °C on a shaker. TBST was then used to wash the wells 4 × before proceeding in one of two ways: (1) biotinylated samples were analyzed by adding 100 µL streptavidin-HRP conjugate in TBST (1:2500 dilution) to the samples before incubating for 3 h at RT on a shaker, or (2) samples without biotin were analyzed by adding 100 µL TBST containing the primary antibody at the manufacturer’s recommendation and incubating for 3 h at RT on a shaker before washing 4 × with TBST. The secondary antibody in 100 µL TBST was then added to the samples following the manufacturer’s recommendation and incubated for 1 h at RT on a shaker. Plates containing either biotinylated or non-biotinylated samples were then washed 5 × with TBST followed by the addition of 100 µL TMB resulting in a blue color change. The reaction was stopped with 100 µL 2 M H_2_SO_4_ after incubating at RT for 0.5–15 min, resulting in a yellow color change that was measured by absorbance at 450 nm. All absorbance measurements were in the linear range. To monitor non-specific binding, negative control wells on the plates included, for example, bound Aβ peptide (Aβ40-HFIP (AS-64128-05), Aβ42-HFIP (AS-64129-05) or conditioned media, then adding all components, streptavidin–horseradish peroxidase and TMB, but without addition of biotin-6E10 or biotin-4G8 antibodies. Some wells were coated with 2.5, 10, 50, 100, 500, and 5000 nM biotin-Aβ (biotin-Aβ40, AS-23512-01; biotin-Aβ42, AS-23523-05) to allow conversion of the OD measurements to concentrations of bound material. Before analysis, the OD from the data were corrected for non-specific binding by subtracting the mean background absorbance for the negative controls. Typically, in control wells incubated on each plate, the background binding is about 10–15% of the maximum binding seen with addition of biotin-peptides or antibodies. Statistical analysis was determined by the GraphPad Prism 8.4.3 software. Data were expressed as the mean ± S.D. Three to five independent experiments were carried out in triplicate for each assay condition.

### Quantitation of Aβ

Aβ ELISAs were carried out according to previous protocols^70–72^ for determining the relative levels of Aβ. Briefly, Aβ1-40 and Aβ1-42 (Aβ40/42) were measured by two-site binding ELISAs using the anti-Aβ42 antibody that is reactive to the C-terminus of Aβ42, or anti-Aβ40 antibody that is reactive to the C-terminus of Aβ40, as the capture antibodies. After incubation with the media and washing the wells, biotinylated-anti-Aβ 6E10 (to Aβ1-16) antibody was added as the detection antibody and then the signal was quantitated using streptavidin–horseradish peroxidase. All values were normalized to data obtained from using the same concentration of samples from the same treatments added to ELISA wells and probed with only biotinylated-anti-Aβ 6E10 antibodies. For Aβ1-x measurements, 6E10 was used as the capture antibody and following incubation with media, biotinylated 4G8 (to Aβ17-24) was added as the detection antibody. For Aβx-42 or Aβx-40, anti-Aβ42 or anti-Aβ40 antibodies, reactive to the C-terminus of Aβ42 and Aβ40, respectively, were used as the capture antibodies and biotinylated-4G8 was added as the detection antibody. All values were normalized to data obtained from using the same concentration of samples from the same treatments added to ELISA wells and probed with only biotinylated-4G8 antibodies.

### MMP2/9 level measurement

The levels of MMP2/9 in the cell culture media were measured using the Invitrogen human MMP2/9 solid-phase sandwich ELISA kits. Briefly, the assay uses a matched antibody pair to measure the amount of the target bound. Samples are added to wells precoated with a target-specific (capture) antibody. The second (detector) antibody is then added and the signal, proportional to the concentration of the target, is detected after addition of a substrate solution.

### PI3K assay

Activated phosphorylated-PI3K p85 + total PI3K p85 in-cell ELISA kit (Abcam) was used according to the recommendations by the manufacturer. Briefly, cells were cultured in 96-well plates then treated as indicated. Following treatment, the cells were fixed, and the wells were then incubated with a primary antibody targeting either total PI3K p85 (recognizes the total level of PI3K p85 proteins regardless of the phosphorylation state) or phosphorylated-PI3K p85 (recognizes p85 PI3K alpha/gamma phospho-tyrosine 467/199). Secondary HRP-conjugated antibodies were then added, and the signal detected after addition of the developing solution. Crystal Violet solution was then added to determine the relative number of cells in each well. Signals for phospho-PI3K and total-PI3K were normalized to cell number then the ratio of phospho-PI3K to total-PI3K for each treatment was determined and plotted.

### AKT assay

The AKT kinase activity assay kit (Abcam) was used to quantitate the activity of AKT according to the manufacturer’s instructions. In brief, the assay is based on a solid phase ELISA. A specific synthetic peptide is used as a substrate for AKT along with a polyclonal antibody that binds the phosphorylated substrate.

### NFκB assay

The NFκB p65 (Phospho/Total) InstantOne sandwich ELISA kit (Invitrogen) was used according to the manufacturer’s recommendations. Signals for phospho (Ser536) and total NFκB were normalized to cell number, then the ratio of phospho (Ser536) to total NFκB for each treatment was determined and plotted.

### MTT assay

The MTT reduction assay (Sigma-Aldrich), used to measure cell viability, was carried out as we reported earlier^50,51,117^. Cells were seeded in 96-well plates as indicated in 200 μL 10% FBS-supplemented media per well and maintained overnight at 95% humidity and 5% CO_2_. After an overnight incubation, the media was replaced with 200 μL serum-free media and the cells were further incubated, without or with different treatments, for 24, 48, or 72 h. The final concentration of DMSO in each well, never exceeded 0.1%. The cells were then incubated for 4 h with MTT (0.5 mg/mL) in the dark. The media was carefully removed and DMSO (100 μL) was added to dissolve the formazan crystals. The absorbance was measured at 570 nm in a plate reader. All absorbance measurements were in the linear range. Untreated cells or wells containing only DMSO and media were used as a positive and negative control, respectively. Statistical analysis was conducted using GraphPad Prism version 8.4.3 for Windows. Significant values were considered at *p* < 0.05 and more significant values at *p* < 0.01, compared with the control.

### Apoptosis assay

Cells were grown as described above then apoptosis was measured using the RealTime-Glo annexin V apoptosis and necrosis assay kit (Promega) according to the instructions provided by the manufacturer. The assay measures phosphatidylserine exposure on the outer leaflet of the cell membrane during apoptosis. The signals were detected using a plate-based multimode reader.

### Immunodepletion

Conditioned media was immunodepleted (ID) according to methods previously described^118^ and our recently published reports^53,57^. ID media was prepared by first growing cells in FBS-supplemented media for 24 h. The cells were then incubated in serum-free media overnight then with the indicated treatments for 72 h. The media was next collected and depleted sequentially from Aβ using first 6E10 antibodies (“Methods” section). The media was then removed and added to wells coated with Aβ40 antibodies, and lastly this media was removed and added to Aβ42 antibodies coated wells. This media, ID of Aβ was then carefully removed and analyzed for the presence of the peptide by ELISA. Significant depletion (95–100%) was observed upon using each of the antibodies employed in this study. The same amount of protein of each sample was analyzed in the experiments.

### Dot blotting

Dot blots were carried out following our previously published procedures^50,51,57^. Cells were grown in 10% FBS-supplemented media overnight in 25 cm^2^ flasks (ThermoFisher) then the cell monolayers were incubated in serum-free media for 24 h. The cells were then treated with 4-MU (600 µM), mouse IgG isotype control with no relevant specificity to a target antigen (mIgG, 5 μg/mL), 5 μg/mL anti-CD44 antibody (5F12) known to be antagonistic towards HA-CD44 molecular interactions, and in combinations. The media was collected and analyzed following incubation of the cell monolayers with the different treatments in serum-free media for 24, 48, and 72 h. After the protein concentrations were determined using the BCA protein assay kit, 3 µL of 600 µg/mL total protein of the conditioned media was spotted onto a nitrocellulose membrane and allowed to dry. Non-specific sites were blocked by soaking the blot for 1 h at RT in a 10 cm Petri dish containing TBST with 5% BSA. The blot was then incubated with the primary antibodies in BSA/TBST overnight at RT following the manufacturer’s recommendation. After washing the membrane with TBST (3 × 5 min), the secondary antibodies conjugated with HRP were added according to the manufacturer's instructions. The membrane was incubated for 30 min at RT then washed 3 × 5 min with TBST and once with TBS for 5 min. Super signal west pico luminol (chemiluminescence) reagent was added to detect the amount of peptide on the membrane, which was then imaged using a Bio-Rad molecular imager, and quantitated with Image J 1.47v software. Distilled water was used as a negative control while purified Aβ peptide was used as a positive control.

### Western blotting

Samples of the cell lysates collected after treatment were analyzed. Attached live cells were harvested and the cell pellet was resuspended in 1 mL lysis buffer consisting of 20 mM Tris/HCl, pH 7.5, 137 mM NaCl, 1% triton X-100, 10% glycerol. Samples were briefly sonicated, centrifuged and the supernatants were stored at − 80 °C until further analysis. The protein concentrations were determined using the BCA protein assay kit. Following methods we reported previously^50^, samples were boiled in 1X SDS, loaded and separated by SDS-PAGE on a 12% gel then transferred to a nitrocellulose membrane. The membrane was blocked in TBST buffer, pH 7.6 containing 5% nonfat milk for 6 h at 4 °C*.* The membrane was then incubated with the specific primary antibody in the blocking buffer, diluted as specified by the manufacturer at RT overnight with gentle shaking. After washing three times with TBST, the membrane was incubated with a HRP labeled secondary antibody in the blocking buffer, diluted according to the manufacturer’s recommendation*.* Subsequent to washing three times in TBST, the blots were developed using super signal west pico luminol (chemiluminescence) reagent and imaged with a Bio-Rad molecular imager.

### SiRNA transfection

Transfections were carried out according to methods reported earlier^51,119^. The day before transfection, cells were seeded at a density of 2 × 10^4^ cells in 25 cm^2^ flasks. Control siRNA, p53 siRNA, AKT siRNA, MMP2 or MMP9 siRNA, were each mixed with Lipofectamine 2000 transfection reagent diluted in Opti-MEM Media (ThermoFisher) for 20 min at RT. The mixtures were then added to the cells to a final concentration of 100 nM for each siRNA and the cells were incubated at 37 °C for 12 h followed by the specific treatments as indicated. The cells were then allowed to incubate from 24 to 72 h at 37 °C. Cells exposed to Lipofectamine 2000 alone were used as a mock control. The media was used to quantitate Aβ levels as described above. Cells collected by trypsinization at the different intervals after transfection were used for Western blotting, while cell viability and apoptosis were measured as described above. Each measurement represents the mean ± S.D. of three-five independent experiments, each performed in triplicate.

### Statistical analysis

The analysis was carried out as we previously reported^51,52,57^. Each experiment in this study was performed in triplicate and repeated a minimum of three times. Statistical values are expressed as the mean ± Standard Deviation (SD). To evaluate the statistical differences, the Mann–Whitney or an ordinary one-way ANOVA followed by Tukey’s post-hoc multiple comparison test was performed. All the statistical tests were two-sided and a *p* value of < 0.05 was considered statistically significant in all cases. GraphPad Prism (GraphPad Software, 8.4.3) was used for the statistical analysis.

## Supplementary Information

Supplementary Information.
