# Supplementary material for: Regulation of amyloid-β levels by matrix metalloproteinase-2/9 (MMP2/9) in the media of lung cancer cells
Source: Sci Rep. 2021 May 6;11:9708. doi: 10.1038/s41598-021-88574-0 (PMC8102533; doi:10.1038/s41598-021-88574-0)
Supplement: Supplementary file 1 — Supplementary Information. [file 41598_2021_88574_MOESM1_ESM.pdf]

Manuscript: Regulation of Amyloid-β Levels by Matrix Metalloproteinase-2/9 (MMP2/9) in the Media of Lung Cancer Cells

Ref: Submission ID 1d1d7fd0-b258-4ec4-bf5e-8cc8af9433cf

Uncropped Images

MMP9 Uncropped Image

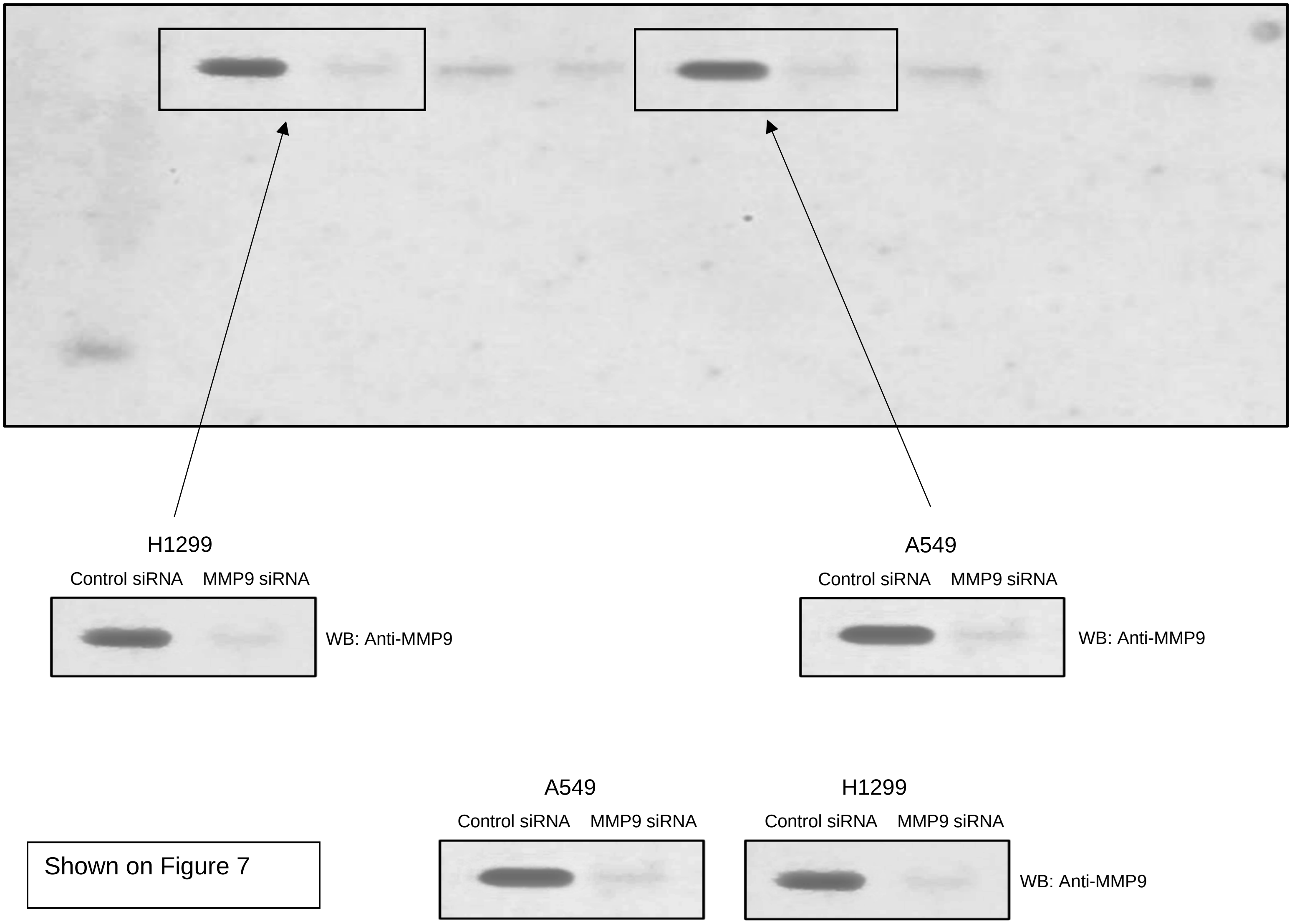

MMP2 Uncropped Image

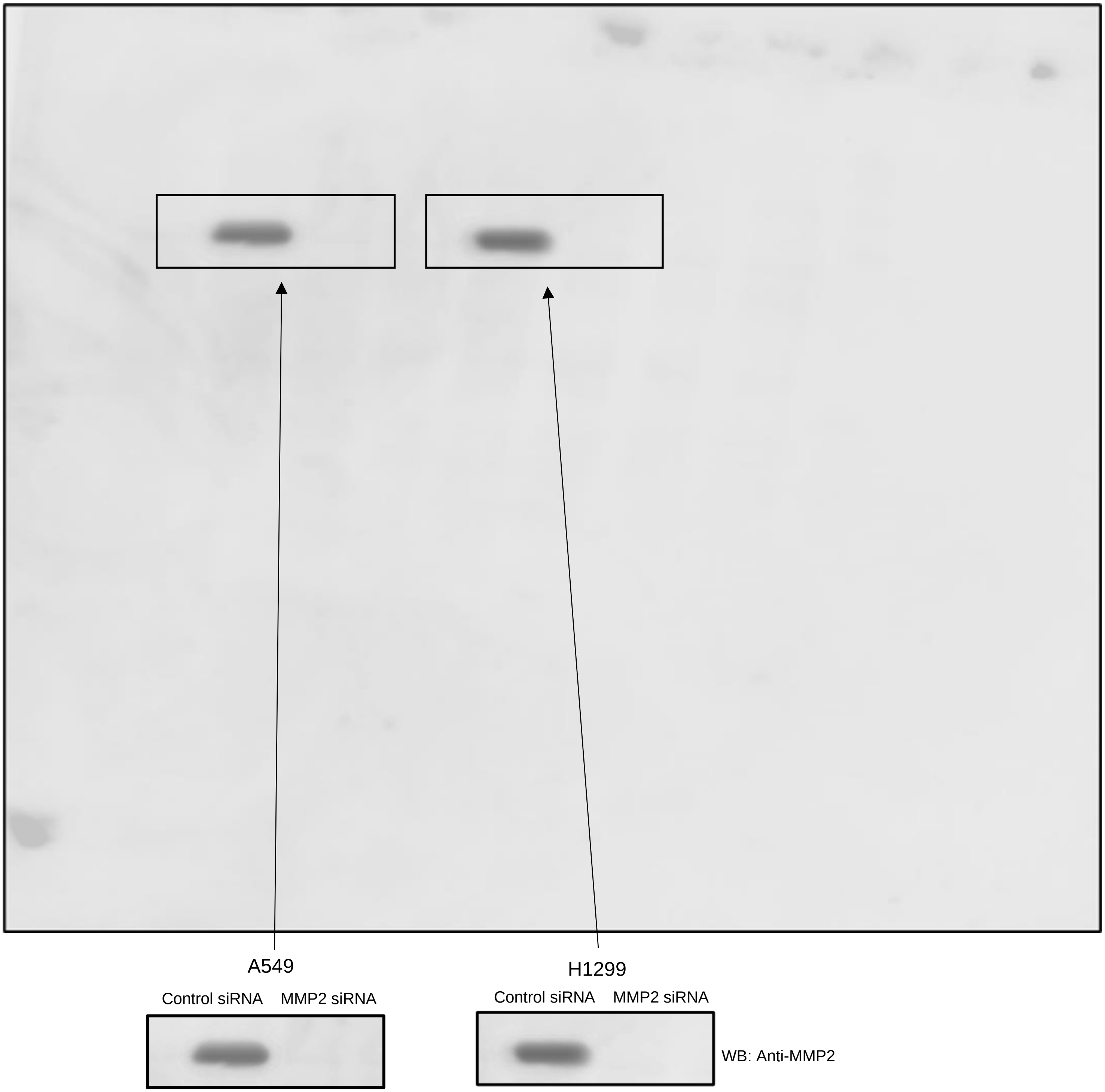

Shown on Figure 7

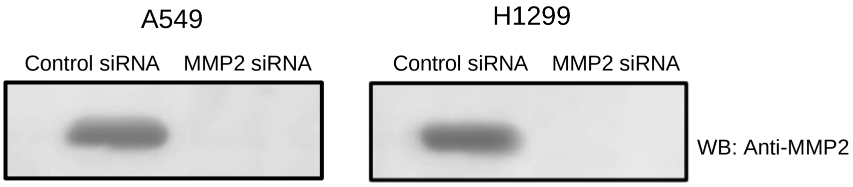

P53 Uncropped Western

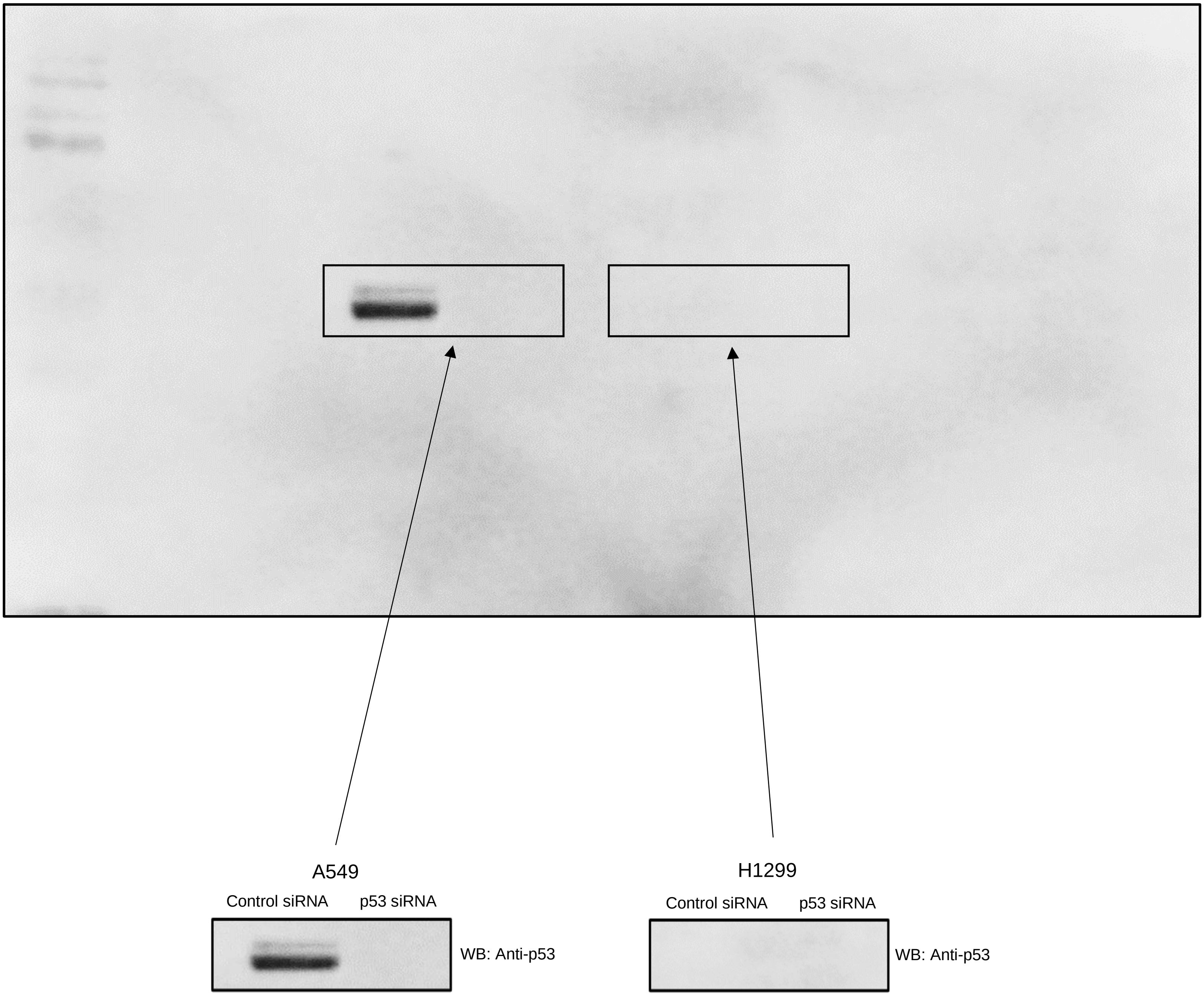

Shown on Figure 7

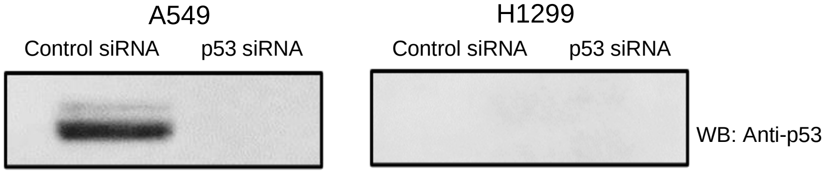

AKT Uncropped Image

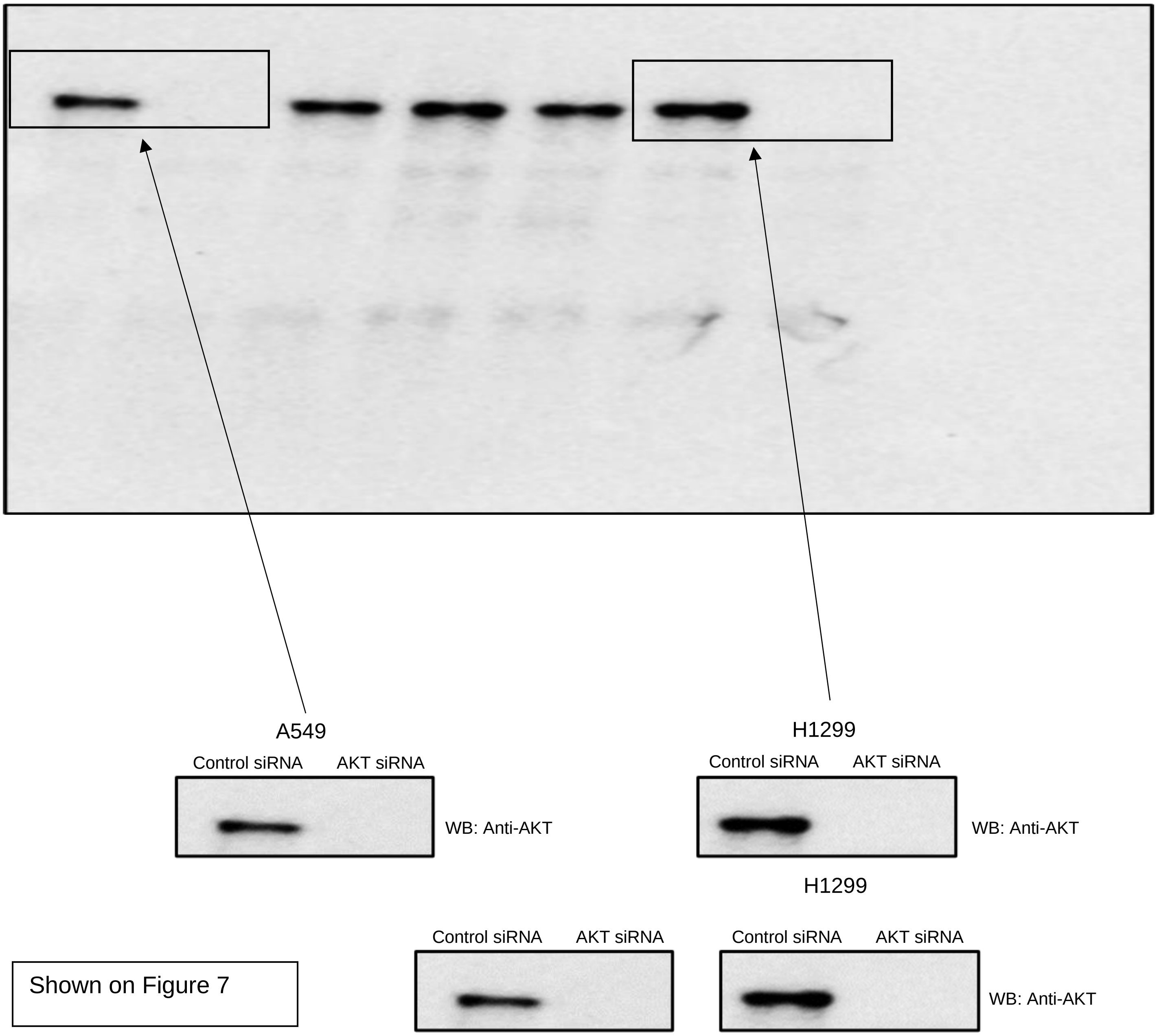

Ponceau Uncropped (A549)

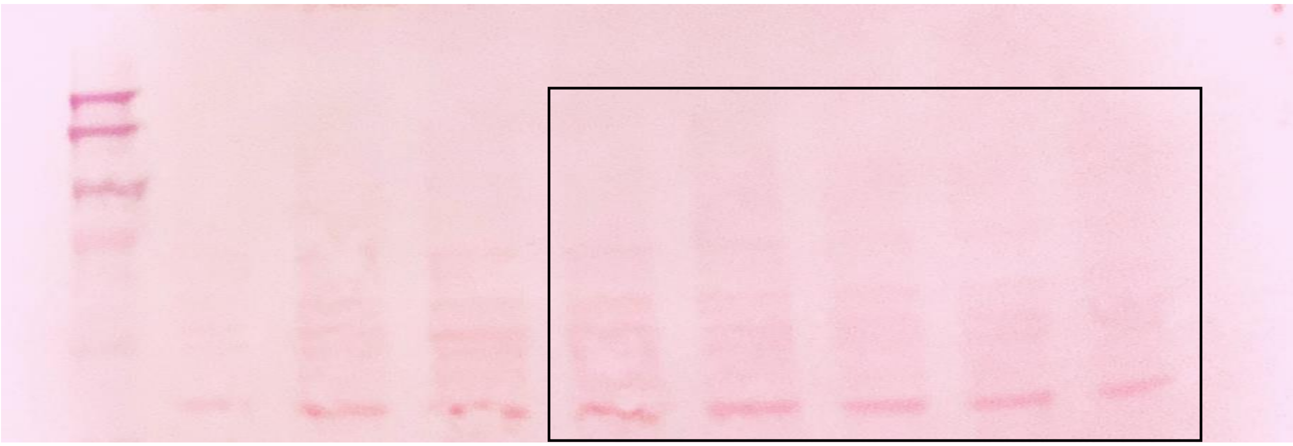

The cropped image on the right was minimized using the upper right-hand corner to produce the image shown in Figure 7.

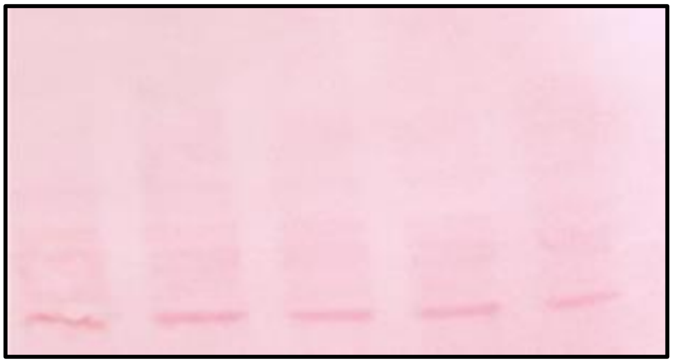

Ponceau (A549)

siRNA: Control MMP9 MMP2 p53 AKT

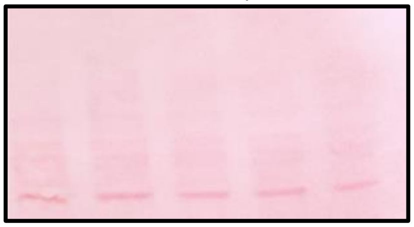

Shown on Figure 7

Uncropped Tubulin (A549)

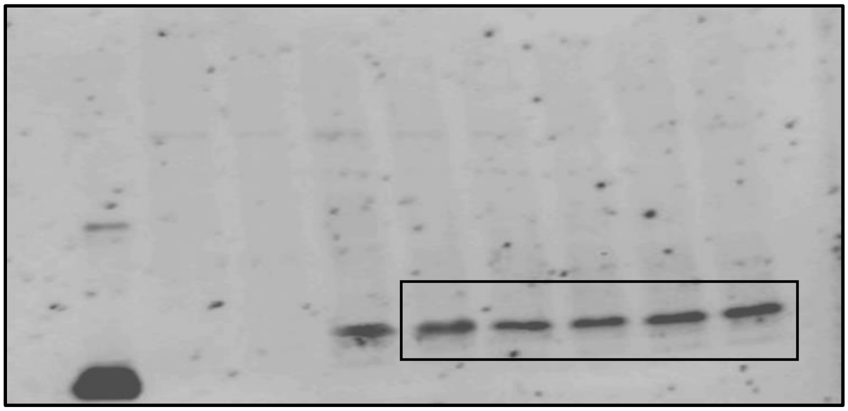

A549

Shown on Figure 7

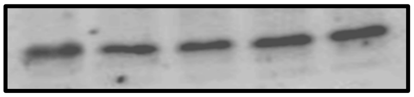

WB: Anti
α- tubulin

Ponceau Uncropped (H1299)

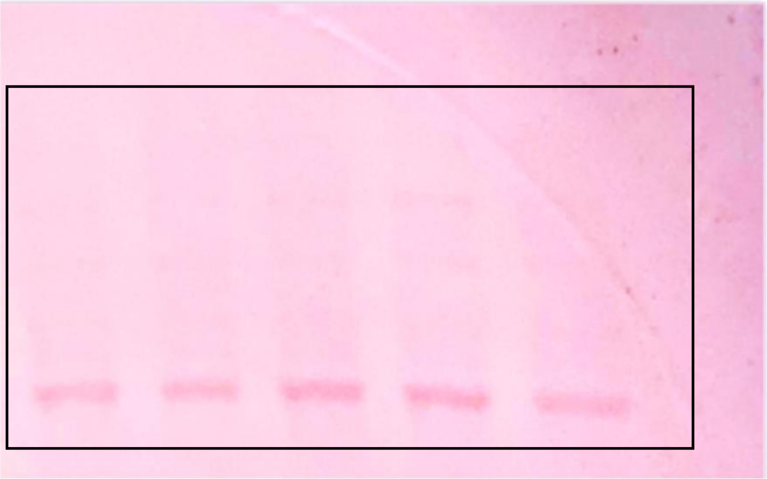

The cropped image on the right was minimized using the upper right-hand corner to produce the image shown in Figure 7.

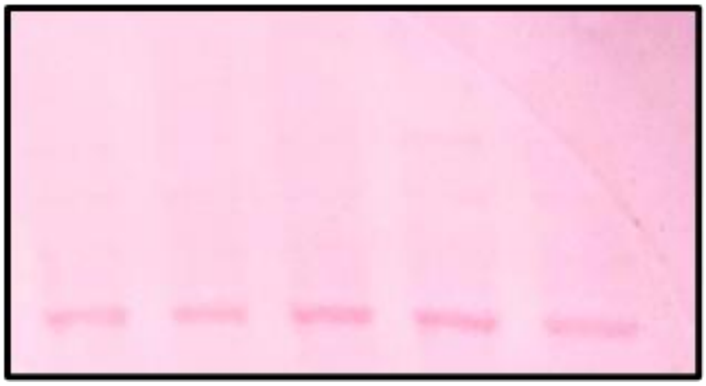

Shown on Figure 7

Ponceau (H1299)

siRNA: Control MMP9 MMP2 p53 AKT

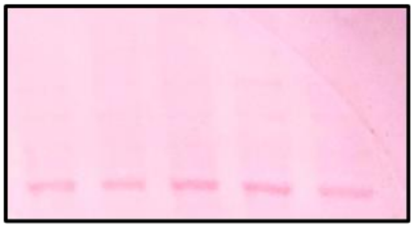

Uncropped Tubulin (H1299)

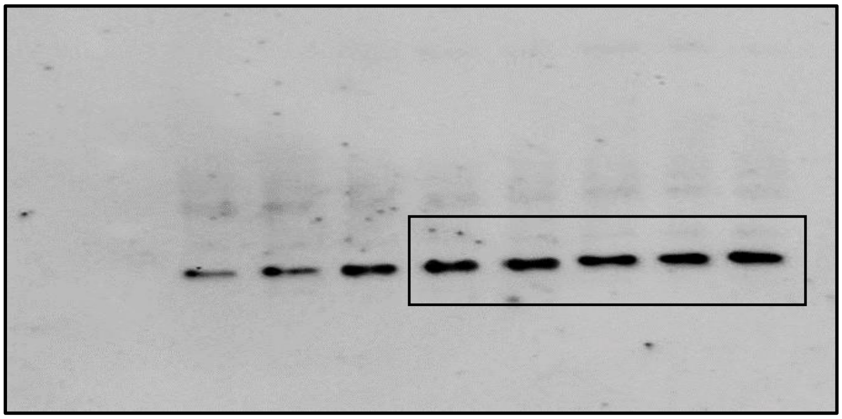

H1299

Shown on Figure 7

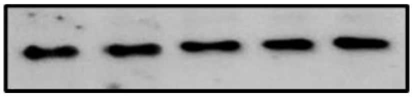

WB: Anti
α- tubulin
